# Assessment of Anhedonia in Adults With and Without Mental Illness: A Systematic Review and Meta-analysis

**DOI:** 10.1001/jamanetworkopen.2020.13233

**Published:** 2020-08-03

**Authors:** Martin Trøstheim, Marie Eikemo, Remy Meir, Ingelin Hansen, Elisabeth Paul, Sara Liane Kroll, Eric L. Garland, Siri Leknes

**Affiliations:** Department of Psychology, University of Oslo, Oslo, Norway; Department of Diagnostic Physics, Oslo University Hospital, Oslo, Norway; Department of Psychology, University of Oslo, Oslo, Norway; Department of Neuroscience, Brown University, Providence, Rhode Island; Department of Diagnostic Physics, Oslo University Hospital, Oslo, Norway; Center for Social and Affective Neuroscience, Department of Biomedical and Clinical Sciences, Linköping University, Linköping, Sweden; Center on Mindfulness and Integrative Health Intervention Development, The University of Utah, Salt Lake City; The University of Utah College of Social Work, Salt Lake City; Department of Psychology, University of Oslo, Oslo, Norway; Department of Diagnostic Physics, Oslo University Hospital, Oslo, Norway

## Abstract

**Importance:**

Anhedonia, a reduced capacity for pleasure, is described for many psychiatric and neurologic conditions. However, a decade after the Research Domain Criteria launch, whether anhedonia severity differs between diagnoses is still unclear. Reference values for hedonic capacity in healthy humans are also needed.

**Objective:**

To generate and compare reference values for anhedonia levels in adults with and without mental illness.

**Data Sources:**

Web of Science, Scopus, PubMed, and Google Scholar were used to list all articles from January 1, 1995 to July 2, 2019, citing the scale development report of a widely used anhedonia questionnaire, the Snaith-Hamilton Pleasure Scale (SHAPS). Searches were conducted from April 5 to 11, 2018, and on July 2, 2019.

**Study Selection:**

Studies including healthy patients and those with a verified diagnosis, assessed at baseline or in a no-treatment condition with the complete 14-item SHAPS, were included in this preregistered meta-analysis.

**Data Extraction and Synthesis:**

Random-effects models were used to calculate mean SHAPS scores and 95% CIs separately for healthy participants and patients with current major depressive disorder (MDD), past/remitted MDD, bipolar disorder, schizophrenia, substance use disorders, Parkinson disease, and chronic pain. SHAPS scores were compared between groups using meta-regression, and traditional effect size meta-analyses were conducted to estimate differences in SHAPS scores between healthy and patient samples. This study followed the Preferred Reporting Items for Systematic Reviews and Meta-analyses (PRISMA) guidelines.

**Main Outcomes and Measures:**

Self-reported anhedonia as measured by 2 different formats of the SHAPS (possible ranges, 0-14 and 14-56 points), with higher values on both scales indicating greater anhedonia symptoms.

**Results:**

In the available literature (168 articles; 16 494 participants; 8058 [49%] female participants; aged 13-72 years), patients with current MDD, schizophrenia, substance use disorder, Parkinson disease, and chronic pain scored higher on the SHAPS than healthy participants. Within the patient groups, those with current MDD scored considerably higher than all other groups. Patients with remitted MDD scored within the healthy range (*g* = 0.1). This pattern replicated across SHAPS scoring methods and was consistent across point estimate and effect size analyses.

**Conclusions and Relevance:**

The findings of this meta-analysis indicate that the severity of anhedonia may differ across disorders associated with anhedonia. Whereas anhedonia in MDD affects multiple pleasure domains, patients with other conditions may experience decreased enjoyment of only a minority of life’s many rewards. These findings have implications for psychiatric taxonomy development, where dimensional approaches are gaining attention. Moreover, the SHAPS reference values presented herein may be useful for researchers and clinicians assessing the efficacy of anhedonia treatments.

## Introduction

Mental disorders are a major cause of disability, affecting 16% to 19% of the world’s population or approximately 1 billion people every year.^1,2^ Traditional diagnostic systems, such as the *Diagnostic and Statistical Manual of Mental Disorders* and the *International Statistical Classification of Diseases,* categorize mental disorders according to constellations of symptoms. However, comorbidity is common, suggesting overlap in symptoms between diagnoses. The National Institute of Mental Health’s Research Domain Criteria Initiative^3^ reconceptualizes psychopathology as varying degrees of impairment across domains and has brought increased attention to transdiagnostic symptoms.

The ability to experience pleasure is essential for well-being,^4^ but is often reduced in mental illness. Anhedonia is defined as a reduced capacity for pleasure^5^ and has been described in major depressive disorder (MDD),^6,7^ bipolar disorder,^6^ schizophrenia,^6,8–11^ substance use disorder (SUD),^12,13^ chronic pain,^14,15^ and Parkinson disease (PD).^16,17^ Despite its presence across numerous psychiatric and neurologic disorders, anhedonia is rarely compared across conditions. Whether anhedonia differs in severity between diagnoses is therefore currently unknown.

Anhedonia is commonly measured using questionnaires,^18^ such as the popular Snaith-Hamilton Pleasure Scale (SHAPS).^19^ The SHAPS is considered “the gold standard for measuring anhedonia in depression,”^18(p27)^ and is also frequently used to assess anhedonia in other patient groups.^13,17,20-33^

The SHAPS consists of 14 confirmatory statements about enjoyable situations typically encountered in daily life cross-culturally (food/drink, interests/pastimes, social interactions, and pleasurable sensory experiences). Respondents to the SHAPS indicate their level of agreement (definitely/strongly agree, agree, disagree, and strongly disagree) with each statement based on their recollection of the last few days. This time frame suggests that the SHAPS is meant to measure a relatively stable state of anhedonia. Responses are summed across items to yield a single anhedonia score.

Despite its popularity, reference values for the SHAPS are lacking and there is no standard scoring method for the questionnaire. Originally, disagreement with more than 2 statements served as a cutoff point between normal hedonic tone and anhedonia.^19^

To compare anhedonia severity across disorders and estimate the threshold for healthy hedonic functioning, we conducted a set of meta-analyses of the numerous publications on studies in which anhedonia symptoms were assessed with the SHAPS. By calculating summary estimates of SHAPS scores (meta-analytic mean and 95% CI) for healthy adults and those with mental illness, we generated reference values for the SHAPS that may guide interpretation of anhedonia severity in future research and clinical settings.

## Methods

### Search Strategy and Selection Criteria

We limited the data material to all articles citing the original SHAPS report by Snaith et al,^19^ identified through Web of Science, Scopus, PubMed, and Google Scholar, and made available between 1995 and 2019. Searches were conducted from April 5 to 11, 2018, and on July 2, 2019. We located the original SHAPS report within each database and used the built-in function of the databases to list and download all articles indexed as citing this report. We also included the original report.^19^ We followed the Preferred Reporting Items for Systematic Reviews and Meta-Analyses (PRISMA) guidelines for reporting of systematic reviews.^34^ A preregistration of this meta-analysis is available in the PROSPERO register.^35^ eAppendix 1 in the Supplement provides the necessary deviations.

Studies were eligible for inclusion if they (1) included original data, (2) used the complete 14-item questionnaire, (3) used 4-point or 2-point scoring of the SHAPS items, (4) assessed SHAPS at baseline or in a no-treatment condition, and (5) did not perform selective recruitment based on SHAPS score. There were no language restrictions.

We categorized samples as healthy if the participants were described as having no current or recent psychiatric and/or medical conditions. Samples were considered to have mental illness if the patients had a verified diagnosis (eg, by structured clinical interview, by qualified professionals, or as a requirement for admission to treatment) according to established criteria (eg, *Diagnostic and Statistical Manual of Mental Disorders* and *International Statistical Classification of Diseases*).

**Figure 1:**
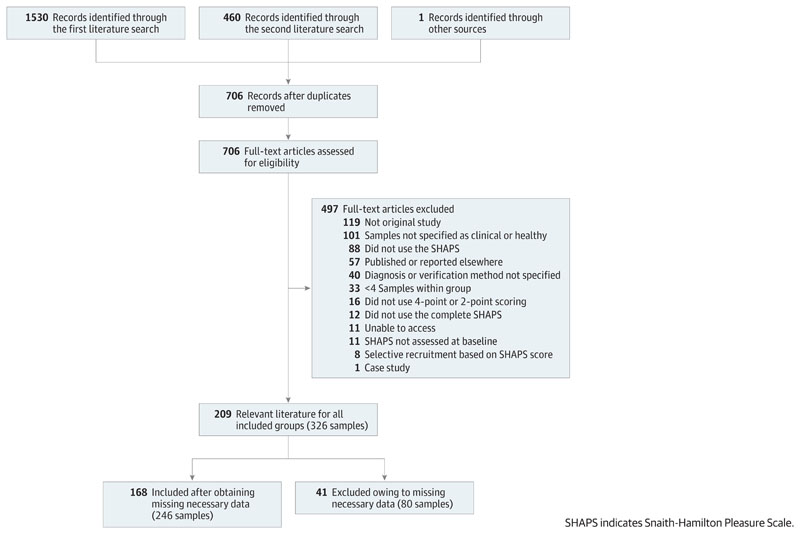
PRISMA Flow Diagram of the Article selection Process

Two researchers examined all the downloaded references using EndNote (Clarivate) (including I.H.), removed duplicates (including I.H.), and evaluated each full-text article independently for inclusion (M.T. and R.M. or I.H.) (Figure 1). Disagreements at this stage were resolved through discussion between the 2 researchers.

### Data Analysis

We did not prespecify which groups to include in the meta-analysis, but decided to evaluate all groups for whom data were available from a minimum of 4 separate samples using the same 2- or 4-point scoring method.^36^ This threshold allowed us to generate nuanced and reliable reference values while keeping the meta-analysis exploratory.

One of us (M.T.) extracted data from all included articles and emailed authors to obtain missing data. For each included sample, the following information was extracted:

1. The total number of participants,
2. The number of female participants,
3. Age (mean and SD),
4. SHAPS information, including scoring method, mean, SD, and the number of participants with anhedonia according to the original cutoff level,
5. Diagnosis
6. Depression score (mean and SD) as measured by various rating scales (eAppendix 1 in the Supplement)
7. General information about the article, including publication year, language, whether it was published in a peer-reviewed journal, and the country of residence for the participants, and
8. The percentage of patients currently receiving medication (MDD, schizophrenia, and PD only).

To produce reliable and representative SHAPS reference values, we aimed to minimize missing data, verify that the questionnaire was sufficiently similar across samples, and ensure minimal diagnostic overlap between groups. The quality assessment therefore calculated (1) the number of samples assessed with a modified SHAPS, (2) the proportion of published data that could be included per group before and after requesting and receiving missing data, and (3) the number of samples with no or any (≥1 participant) comorbidity with MDD, psychotic symptoms or disorders, SUD, and anxiety disorders.

Since different iterations of 2-point (eg, 0-1, 1-0) and 4-point (eg, 1-4, 4-1, 0-3, and 3-0) SHAPS scoring formats have been reported,^24,37,38^ we recalculated scores from some studies to conform to either a 0 to 1 (1, disagree or strongly disagree) or 1 to 4 scoring method (4, strongly disagree). While the range of possible SHAPS scores differed for the 2-point (0-14) and 4-point (14-56) scales, higher values indicated greater anhedonia symptoms in both cases.

### Statistical Analysis

All analyses were performed using random-effects models implemented in the metafor package^39^ in R statistical software, version 3.5.2.^40^ We used the DerSimonian-Laird method^41^ for estimating the between-studies variance component (*T* ^2^ in each random-effects model and calculated 95% CIs using the critical *z* value at a = .05. Results were considered statistically significant if *P* < .05, as determined with 2-tailed, unpaired testing. Multiple testing is common yet seldom addressed in meta-analyses,^42,43^ and consensus on how to account for multiple testing is lacking.^44,45^ Results are reported herein without adjustments for multiple testing.

The primary set of meta-analyses produced and compared point estimates of the mean SHAPS scores for each included group. Separate random-effects models were computed for each included group using SHAPS scores of individual samples as input. These meta-analyses were performed separately for studies using 4-point and 2-point SHAPS scoring formats. We used meta-regression to compare groups.

The second set of meta-analyses consisted of traditional effect size meta-analyses of standardized differences in SHAPS scores between healthy groups and those with mental illness. We used Hedges *g* ^46^ as the effect size measure and meta-regression to compare effect sizes between groups.

We performed additional meta-regressions to assess the importance of age, sex, general depression severity, medication status (current MDD, schizophrenia, and PD only), and drug use status (SUD only) for SHAPS scores. eAppendix 1 in the Supplement provides more details and analytic considerations, including sensitivity analyses (eTables 1-6 in the Supplement) and a small-scale meta-analysis of individual SHAPS items.

## Results

The final data material contained 168 studies assessing SHAPS scores in 246 samples (Figure 1; eTable 7 in the supplement) of healthy participants and patients with current and past MDD, bipolar disorder, schizophrenia, SUD, PD, and chronic pain (N = 16 494; 8058 [49%] female; 7298 [44%] male; 1138 [7%] missing accurate sex data; and age range, 13-72 years). eTable 8 in the Supplement provides group characteristics. Data on anxiety-related and eating disorders were not included in the meta-analysis owing to limited availability but are presented in eTable 9 in the Supplement.

### Quality Assessment

Risk of bias owing to modifications of the SHAPS was low, as the questionnaire was largely invariant across studies. Fifty-three samples (21%) used non-English translations of the SHAPS. Other minimal modifications occurred in only 4 samples (2%)^47–49^ (eAppendix 2 in the supplement).

Before we contacted authors, necessary SHAPS data were available for only 13% to 80% (mean, 33%) of the identified samples for each included group (Figure 1; eTable 10 in the Supplement). After obtaining missing data, we were able to include 70% to 100% (mean, 75%) of the identified samples. This addition reduced the risk of publication bias and bias due to selective reporting of SHAPS scores.

There was little diagnostic overlap between the MDD, schizophrenia, and SUD groups (eTable 11 in the Supplement). Information about co-occurring psychiatric disorders was often lacking for PD samples, and comorbidity with anxiety disorders was rarely reported for any group. The low comorbidity allowed us to largely isolate the anhedonia severity associated with each diagnosis.

### Meta-analyses

**Figure 2:**
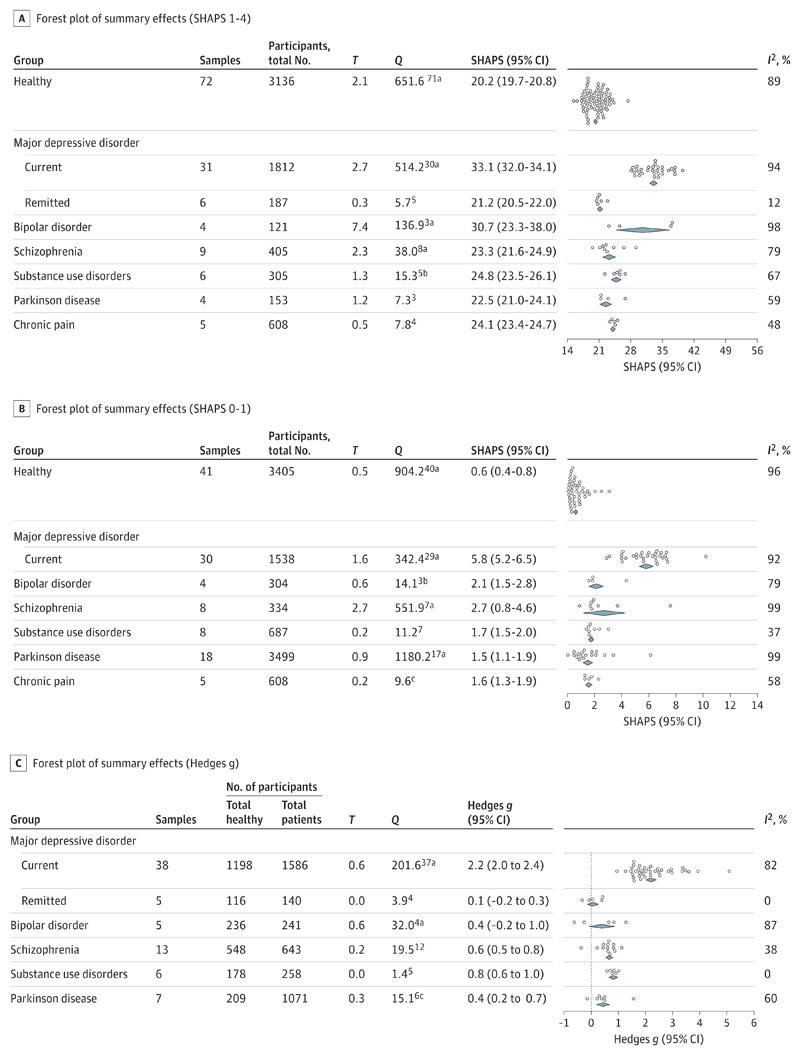
Sets of Meta-analysis of Snaith-Hamilton Pleasure Scale (SHAPS) Scores Across Groups A, SHAPS scores from studies using 1- to 4-point scoring showing significantly higher anhedonia in all patient groups compared with healthy individuals. B, SHAPS scores from studies using the original 0- to 1-point scoring method replicates the pattern found in studies using 4-point scoring. Note that except for chronic pain, there was no overlap between studies included in A and B. C, Effect sizes based on studies reporting scores from patients and controls, according to both scoring methods. Diamonds indicate mean and 95% CI. White dots indicate individual sample means. I^2^ indicates the amount of variation between samples that is due to heterogeneity rather than chance; Q, Cochran Q test; and T, estimated between-samples SD. ^a^ *P* < .001. ^b^ *P* < .01. ^c^ *P* < .05.

With the 1 to 4 scoring format (Figure 2A), SHAPS scores for individuals with current MDD (mean, 33.1 points; 95% CI, 32.0-34.1 points), schizophrenia (mean, 23.3 points; 95% CI, 21.6-24.9 points), SUD (mean, 24.8 points; 95% CI, 23.5-26.1 points), PD (mean, 22.5 points; 95% CI, 21.0-24.1 points), and chronic pain (mean, 24.1 points; 95% CI, 23.4-24.7 points) were significantly higher than those of the healthy group (mean, 20.2 points; 95% CI, 19.7-20.8 points). Table 1 provides group comparisons. These findings suggest that anhedonia occurs in these conditions. Compared with current MDD, SHAPS scores were nevertheless significantly lower in all other types of mental illness. SHAPS scores in remitted MDD (21.2; 95% CI, 20.5-22.0) were comparable to those of healthy samples. Thus, anhedonia severity differed between diagnoses. This pattern was replicated with 0 to 1 scoring (Figure 2B; Table 1) despite no overlap of included samples for any group except chronic pain. On average, healthy individuals disagreed with 1 SHAPS item, patients with MDD disagreed with 6 items, and the groups with other types of illness disagreed with 3 or fewer items. Simplified reference values based on these results are available in Table 2.

Meta-analyses of effect sizes (Figure 2C) were conducted on studies using either scoring method and including data from both patients and healthy controls. Again, SHAPS scores for patients with current MDD were significantly above levels in healthy individuals (Hedges *g*, 2.2; 95% CI, 2.0-2.4), schizophrenia (Hedges *g*, 0.6; 95% CI, 0.5-0.8), SUD (Hedges *g*, 0.8; 95% CI, 0.6-1.0), and PD (Hedges *g*, 0.4; 95% CI, 0.2-0.7), but not in remitted MDD (Hedges *g*, 0.1; 95% CI, -0.2 to 0.3). SHAPS scores were significantly higher in current MDD compared with any other group (Table 1). Although no formal subgroup analyses could be performed for the bipolar disorder group, data from both scoring methods and the effect size analysis suggested markedly higher SHAPS scores in individuals with depression (Hedges *g*, 1.3; 95% CI, 0.8-1.8) compared with mania (Hedges *g*, -0.6; -1.2 to 0.0) and euthymia (Hedges *g*, -0.3; 95% CI, -0.9 to 0.3).

Neither age nor sex ratio could explain the observed differences in SHAPS scores between healthy groups and those with mental illness in most of the analyses (eTable 12 and eTable 13 in the supplement). Results from meta-regressions adjusting for general depression severity varied across scoring methods and analyses (eTable 14 in the Supplement), consistent with the notion that anhedonia in schizophrenia, SUD, PD, and chronic pain is unlikely to result solely from comorbid depression.

Within groups, age and sex differences in SHAPS scores were generally small and/or nonsignificant (eTable 15 and eTable 16 in the supplement). SHAPS scores in current MDD, schizophrenia, and PD did not significantly vary with the percentage of patients receiving medications at the time of assessment (eTable 17 in the supplement). Moreover, SHAPS scores in SUD samples categorized as currently abstinent (n = 258) were comparable to scores in individuals currently using substances (n = 429;B = -0.19; SE, 0.27; *P* = .48).

## Discussion

To our knowledge, it has not been possible previously to compare the degree of anhedonia symptom load across diagnoses, despite the extensive data available in the literature. We used a meta-analytic approach to generate suggested reference values for the level of anhedonia in adults with and without mental illness based on SHAPS scores from 16 494 people. While anhedonia scores were significantly increased in current but not remitted MDD, schizophrenia, SUD, PD, and chronic pain compared with healthy participants, we found evidence for substantially higher anhedonia in ongoing MDD compared with other types of illness. This pattern replicated across scoring methods for the SHAPS and was consistent across point-estimate and effect size analyses.

Our findings apparently support the clinical association between anhedonia and schizophrenia, SUD, PD, and chronic pain.^6,7,12,17^ The observed variability in anhedonia severity across conditions is consistent with Research Domain Criteria’s dimensional approach to mental disorders. Anhedonia in some conditions may be qualitatively as well as quantitatively distinct from anhedonia during major depression. The high SHAPS scores support the hypothesis that anhedonia in MDD affects multiple domains of pleasure (eg, food/drink, pastimes/hobbies, social, and physical). Patients with MDD reported that they would not enjoy, on average, 6 of the 14 listed everyday rewards. In contrast, healthy participants reported, on average, 1 unenjoyable SHAPS item, and the groups with other types of mental illness all averaged below 3 of the items.

**Figure 3:**
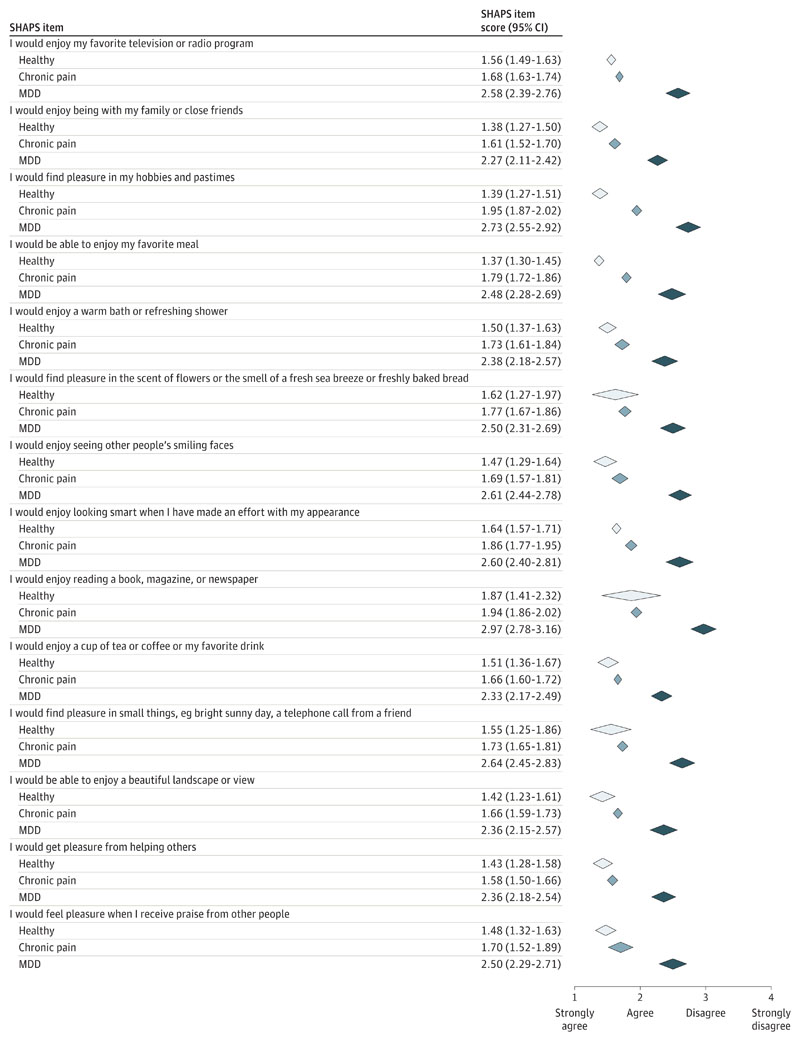
Exploratory item-Level Meta-analysis To test whether patients with a specific mental health diagnosis typically experience anhedonia for the same subset of pleasures, we conducted an exploratory meta-analysis of raw, item-level data from 376 healthy volunteers, 64 patients with major depression, and 487 chronic pain patients (for details, see eAppendix 1 in the Supplement). Item-level data for other groups were not available to us at the time of writing. Diamonds indicate mean and 95%CI. SHAPS indicates Snaith-Hamilton Pleasure Scale.

An item-level meta-analysis of available data from individuals with MDD, chronic pain, and healthy volunteers showed that this pattern appears to be consistent (Figure 3), with modest increases in anhedonia for all items in chronic pain. Similarly, patients with MDD scored consistently higher on every SHAPS item. Thus, at the group level, we found no support for the notion that anhedonia in patients with chronic pain or MDD is associated with specific impairments, such as anosmia. Instead, MDD and chronic pain may uniformly dampen people’s enjoyment of life.

Despite reported behavioral and neural reward impairments in remitted MDD,^50–53^ we found no demonstrable anhedonia in this group. Instead, people with remitted MDD reported projected enjoyment of rewards that is comparable to that of healthy individuals. Similarly, mania and euthymia states in bipolar disorder were associated with markedly lower SHAPS scores than depressed states, consistent with the presence of hyperhedonia (increased enjoyment of rewards^54^) during nondepressed stages. Together, these cross-sectional data support the view of anhedonia as a relatively stable yet reversible state in depression and suggest that anhedonia fluctuates together with some other symptoms of depression. Longitudinal data are needed to explore phase dependencies of anhedonia in depression and evaluate which other depression symptoms are temporally associated with anhedonia.

The indications of reversibility suggest its utility for the development of therapies for anhedonia, which is often considered a difficult symptom to treat.^55,56^ New psychotherapies focusing on savoring and increasing positive affect are emerging,^57^ with demonstrable effects on brain reward processing.^58^ Initial studies reported antianhedonic effects of antidepressant medications, as discussed by Cao et al,^59^ yet better-controlled investigations, such as that conducted by Krystal et al,^60^ are needed. The reference values provided herein may be useful when the efficacy of new and existing treatments of anhedonia is assessed.

Anhedonia is a key symptom thought to differentiate depression from anxiety disorders.^61^ While there were insufficient data to include anxiety disorders in the current meta-analyses, the 3 available studies on posttraumatic stress disorder reported SHAPS scores comparable to severe anhedonia levels in current MDD.^62–64^ Only modest anhedonia as measured by the SHAPS has been reported in individuals with obsessive-compulsive disorder.^65,66^ Despite theoretical interest in the role of anhedonia and reward functioning for eating disorders,^67–69^ we could retrieve SHAPS scores from only 2 studies. These scores were consistent with mild anhedonia in anorexia nervosa.^70,71^

Dysfunction in the mesolimbic dopamine system and its interactions with the endogenous opioid system have been proposed as a central mechanism underlying anhedonia.^12,72^ Recent evidence suggests that there are similarities in the genetic and neural underpinnings of anhedonia across multiple disorders.^73^ It is unclear whether differences in anhedonia severity across conditions observed herein with the SHAPS reflect different physiologic pathways or distinct levels of disruption of the same underlying mechanisms.

### Limitations

This study has limitations. The SHAPS literature consists primarily of smaller-scale studies of patients without comorbidities and is therefore likely not representative of the entire patient populations. Accordingly, bias in representativeness was not formally assessed.^74^ Conversely, these reference values may be more indicative of the levels of anhedonia specifically associated with each disorder in isolation, and therefore useful in improving discriminant validity of psychiatric taxa in taxometric investigations and future nosologic efforts. Large-scale epidemiologic studies are needed to produce anhedonia severity estimates that generalize to the larger patient populations in which diagnostic comorbidity is more common. This meta-analysis operationalized anhedonia as scores on the SHAPS and results may not generalize to other anhedonia questionnaires or other facets of reward processing outlined in the Research Domain Criteria framework.

Reference values for some of the smaller groups (eg, schizophrenia, SUD, and PD)may be less reliable than those for the larger groups (healthy and current MDD). However, the similar pattern of results found across the independent samples scored with the 2- and 4-point formats speaks to the stability, generalizability, and statistical coherence of the present results.

Smoking is common in patients with mental illness^75^ and has bidirectional associations with anhedonia.^76,77^ Owing to limited data and inconsistent reporting across studies, we were unable to evaluate potential moderating effects of smoking behavior on SHAPS scores. For the same reason, we were able to assess the effect of medication status on anhedonia only in MDD, schizophrenia, or PD and not the effects of specific drugs. Moderating effects of age and sex were estimated as modest.

## Conclusions

The results of this meta-analysis suggest that anhedonia, as measured by the SHAPS,differs quantitatively across conditions typically associated with this symptom. While modest anhedonia was seen in patients with schizophrenia, SUD, PD, and chronic pain, studies have consistently reported more severe anhedonia in patients with current MDD. We recommend that, for clarity and ease of comparison across samples,researchers and clinicians report SHAPS scores using both the 2- and 4-point scoring methods applied here,taking care to ensure that higher scores indicate anhedonia.

## Supplementary Material

Supplementary

## Figures and Tables

**Table 1:** Between-Groups Comparisons Using Meta-regression


	Scoring						
	1-4			0-1			Effect size comparisons		
Comparison	B (SE)	*z* Value	*P* value	B (SE)	*z* Value	*P* value	B (SE)	*z* Value	*P* value

Healthy vs MDD								

Current	12.83 (0.54)	23.72	<.001	5.11 (0.19)	27.41	<.001	NA	NA	NA

Remitted	0.99 (0.95)	1.04	.30	NA	NA	NA	NA	NA	NA

MDD (remitted) vs MDD (current)	11.84(1.24)	9.55	<.001	NA	NA	NA	2.11 (0.32)	6.55	<.001

Healthy vs SCZ	3.01 (0.85)	3.55	<.001	2.13 (0.28)	7.52	<.001	NA	NA	NA

SCZ vs MDD (current)	9.78 (1.12)	8.74	<.001	3.10 (0.86)	3.61	<.001	1.59 (0.19)	8.48	<.001

Healthy vs SUD	4.64 (0.96)	4.81	<.001	1.21 (0.23)	5.23	<.001	NA	NA	NA

SUD vs MDD (current)	8.17 (1.26)	6.47	<.001	3.92 (0.51)	7.77	<.001	1.39 (0.28)	4.88	<.001

Healthy vs PD	2.50(1.21)	2.06	.04	0.81 (0.20)	4.10	<.001	NA	NA	NA

PD vs MDD (current)	10.22 (1.56)	6.55	<.001	4.27 (0.32)	13.52	<.001	1.74 (0.27)	6.51	<.001

Healthy vs chronic pain	4.00 (0.97)	4.11	<.001	0.99 (0.27)	3.67	<.001	NA	NA	NA

Chronic pain vs MDD (current)	8.82 (1.25)	7.04	<.001	4.16 (0.62)	6.73	<.001	NA	NA	NA


1-4 and 0-1 scoring: B and SE are on the same scale as the Snaith-Hamilton Pleasure Scale. Effect size: B and SE are on the same scale as Hedges g. Abbreviations: MDD, major depressive disorder; PD, Parkinson disease; SCZ, schizophrenia; SUD, substance use disorders.

**Table 2:** SHAPS Reference Values

	Scoring, mean (SD) [range]^a^			
Group	1-4 (14-56)	0-1 (0-14)	Anhedonia mean (range), %^b^	Effect size, mean (SD) [range]^c^
Healthy	20.2 (2.1) [15.4-27.4]	0.6 (0.5) [0.1-3.1]	14 (0-15)	NA

Major depressive disorder				

Current	33.1 (2.7) [28.2-39.5]	5.8 (1.6) [2.9-10.2]	62 (35-87)	2.2 (0.6) [0.9-5.1]

Remitted	21.2 (0.3) [20.4-22.9]	NA	NA	0.1 (0.0) [-0.3 to 0.4]

Schizophrenia	23.3 (2.3) [19.6-29.2]	2.7 (2.7) [0.9-7.6]	23 (NA)	0.6 (0.2) [-0.4 to 1.1]

Substance use disorders	24.8 (1.3) [22.3-26.8]	1.7 (0.2) [1.4-3.0]	31 (19-55)	0.8 (0.0) [0.6-1.0]

Parkinson disease	22.5 (1.2) [21.4-26.8]	1.5 (0.9) [0.0-6.1]	25 (5-46)	0.4 (0.3) [-0.1 to 1.6]

Chronic pain	24.1 (0.5) [23.4-25.1]	1.6 (0.2) [1.3-2.3]	23 (14-34)	NA

Abbreviations: NA, not applicable; SHAPS, Snaith-Hamilton Pleasure Scale.

^a^ Higher scores indicate greater anhedonia. Model-based percentile cutoffs for healthy participants in the 1- to 4-point scoring format: 15.3 (1st), 18.8 (25th), 20.2 (50th), 21.6 (75th), and 25.1 (99th). Model-based percentile cutoffs for healthy participants in the 0- to 1-point scoring format: 0.0 (1st), 0.3 (25th), 0.6 (50th), 0.9 (75th), and 1.8 (99th). These percentile cutoffs indicate which SHAPS scores a certain percentage of healthy participants score below.

^b^ Anhedonia indicates the percentage of people scoring above the original SHAPS cutoff (>2 with 0-1 scoring)^19^ in the small subset of samples for which this information is available (healthy: n = 3, major depressive disorder [current]: n = 3, schizophrenia: n = 1, substance use disorders: n = 7, Parkinson disease: n = 8, and chronic pain: n = 5).

^c^ Effect sizes (Hedges *g*) indicate the standardized difference between the healthy group and a patient group and allow for comparisons with other measurements.

## Key Points

### Question

Does anhedonia severity differ among patients with different types of mental illness typically associated with this symptom, and what is considered healthy hedonic functioning?

### Findings

In this systematic review and meta-analysis of 168 studies including more than 16 000 participants, anhedonia as measured by the Snaith-Hamilton Pleasure Scale was significantly elevated in patients with major depressive disorder, schizophrenia, substance use disorders, Parkinson disease, and chronic pain. Compared with ongoing major depressive disorder, all other patient groups displayed significantly lower anhedonia.

### Meaning

The findings of this metaanalysis provide a possible set of reference values for anhedonia severity across healthy populations and those with mental illness; these results may have utility for researchers and clinicians evaluating new and existing treatments for anhedonia.
