# Supplementary material for: Assessment of Anhedonia in Adults With and Without Mental Illness: A Systematic Review and Meta-analysis
Source: JAMA Netw Open. Author manuscript; Available in PMC 2020 Oct 9. (PMC7116156; doi:10.1001/jamanetworkopen.2020.13233)
Supplement: Supplementary [file EMS95124-supplement-Supplementary.pdf]

Supplementary Online Content

Trøstheim M, Eikemo M, Meir R, et al. Assessment of anhedonia in adults with and without mental illness: a systematic review and meta-analysis. *JAMA Netw Open*. 2020;3(8):e2013233. doi:10.1001/jamanetworkopen.2020.13233

eAppendix 1. Methods

eAppendix 2. Results

eTable 1. Effect Size Meta-analyses With the PM Estimator of the Between-Studies Variance

eTable 2. Between-Groups Comparisons Using Meta-Regression With the PM Estimator of the Between-Studies Variance

eTable 3. Effect Size Meta-analyses With the REML Estimator of the Between-Studies Variance

eTable 4. Between-Groups Comparisons Using Meta-Regression With the REML Estimator of the Between-Studies Variance

eTable 5. Effect Size Meta-analyses With Random Effect at the Article Level

eTable 6. Between-Groups Comparisons Using Meta-Regression With Random Effect Added at the Article Level

eTable 7. Sample Details for All Included Groups

eTable 8. Group Characteristics

eTable 9. Sample Details for the Anorexia Nervosa, Obsessive-Compulsive Disorder and Posttraumatic Stress Disorder Groups

eTable 10. Completeness of Necessary Data for Each Included Group

eTable 11. Reporting of Comorbidity for Clinical Samples

eTable 12. Between-Groups Comparisons Adjusting for Age

eTable 13. Between-Groups Comparisons Adjusting for Percent Female Participants

eTable 14. Between-Groups Comparisons Adjusting for Depression Severity

eTable 15. The Contribution of Age to SHAPS Scores

eTable 16. The Contribution of Percent Female Participants to SHAPS Scores

eTable 17. The Contribution of Percent Medicated Patients to SHAPS Scores

eReferences

This material has been provided by the authors to give readers additional information about their work.

eAppendix 1. **Methods**

The main analysis was conducted in April 2019 and updated with new data in autumn 2019. Preliminary results have been reported in M.T.'s master thesis[1] and presented at the European Behavioural Pharmacology Society Biannual Meeting[2] and Vettreseminaret[3] in 2019.

1.1 Data extraction and preprocessing

We extracted depression mean and standard deviation as measured by the following rating scales: Beck Depression Inventory (BDI),[4,5] Center for Epidemiologic Studies Depression Scale (CES-D),[6] Depression Anxiety Stress Scales (DASS),[7] Hamilton Rating Scale for Depression (HAM-D),[8] Montgomery-Åsberg Depression Rating Scale (MADRS),[9] Mood and Feelings Questionnaire (MFQ),[10] the depression subscale of the Hospital Anxiety and Depression Scale (HADS),[11] the Inventory of Depressive Symptomatology (IDS),[12] the Quick Inventory of Depressive Symptomatology (QIDS),[13] the Bech-Rafaelsen Melancholia Scale (BRMS),[14] the General Distress: Depressive Symptoms and Anhedonic Depression subscales of the Mood and Anxiety Symptom Questionnaire (MASQ),[15] the Geriatric Depression Scale (GDS),[16] Zung Self-Rating Depression Scale (SDS),[17] Self-Rating Questionnaire for Depression (SRQ-D),[18] the depression item on the Short Parkinson's Evaluation Scale (SPES),[19] and the Calgary Depression Scale (CDS).[20]

To facilitate comparison across samples and between groups, we created a common depression severity variable. For each measure of depression, we rescaled each sample mean score according to the highest obtainable score on each particular instrument. The resulting scores expressed depression severity in percentage of the maximum score and could therefore range from 0-100, with higher scores indicating greater severity and/or more symptoms of depression. For samples with more than one depression measure reported, we then averaged this percentage score across all available measures of depression.

1.2 Comparisons of group characteristics

We used two-tailed z-tests to assess group differences in age, percentage of female participants, general depression severity, and percentage of anhedonic participants according to the original Snaith-Hamilton Pleasure Scale (SHAPS) cut-off .[21]

1.3 Quality assessment

The descriptive risk of bias assessment followed best practice recommendations by The Cochrane Collaboration,[22] instead of our preregistered plan,[23] since the latter could not fully distinguish between quality of reporting and conduct.

Because we analyzed baseline SHAPS data obtained before any intervention, standard assessment tools for risk of bias in randomized controlled trials were inappropriate.[24]

1.4 Meta-analyses

Heterogeneity refers to the variation in the true effects underlying each study in the meta-analysis.[25] Different heterogeneity estimates are reported with meta-analyses to give an overview of the spread of the study effects. *Cochran's Q* is used to test the null-hypothesis that all studies share the same underlying effect. A significant *Cochran's Q* suggests that there is variation in the observed study effects that cannot be explained by sampling error. I^2 complements *Cochran's Q* by indicating "the percentage of total variation across studies that is due to heterogeneity rather than chance."[26(p558)] Neither *Cochran's Q* nor I^2 allow us to evaluate the spread of the study effects on the same scale as the outcome measure. For this, we calculated T (i.e. the square root of T^2), which indicates the between-studies standard deviation of the observed study effects.[25] For moderator analyses, we also calculated R^2, which indicates the percentage of the total heterogeneity that is explained by the moderator(s).[27]

1.5 Sensitivity analyses

To test whether the results from our meta-analyses were dependent on the choice of methods for estimating the between-studies variance, we repeated the primary analyses using other recommended tau^2 estimators for continuous outcomes, including the Paule-Mandel (PM) and the restricted maximum likelihood (REML) methods.[28] Because data for some samples were from the same articles, we tested the potential clustering effects by repeating analyses with an added random effect at the article level.[29]

© 2020 Trøstheim M et al. *JAMA Network Open.*

1.6 Additional meta-regressions

Due to differences in age and gender distribution between the healthy and clinical groups (eTable 8), we performed additional meta-regression adjusting for age. For point-estimate analyses, this meant adding both group and age or gender (i.e. percent female participants) as predictors. For effect size analyses, we adjusted for the difference in age or gender between healthy and patient samples when estimating the summary effect sizes.

To assess whether elevated anhedonia in schizophrenia (SCZ), substance use disorders (SUD), Parkinson's disease (PD) and chronic pain compared could be explained by general depression severity, we conducted meta-regressions adjusting for depression scores (point-estimate analyses) or the difference in depression scores (effect-size analyses).

We also performed meta-regressions to address whether SHAPS scores in major depressive disorder (MDD), schizophrenia and PD samples were related to the percentage of patients ON medications at the time of assessment.

Finally, we used meta-regression to assess the importance of drug use status (dummy-coded: current use = 0, abstinent = 1) for anhedonia in SUD. Due to insufficient data based on four-point scoring, only data based on two-point scoring were considered in this analysis (current use: $k = 4$, $n = 429$; abstinent: $k = 4$, $n = 258$). The duration of abstinence in these samples ranged from ~2 weeks to ~1 year.

While we intended to assess the contribution of unpublished studies, non-English articles and SHAPS modifications to the reference values generated in this meta-analysis (see preregistration[23]), there was too little variation in these variables within each group for the analyses to be feasible.

1.7 Item-level group comparison

To test whether patients within a clinical group typically experience anhedonia for the same subset of pleasures, we conducted an exploratory meta-analysis of raw, item-level data. For this item-level analysis, we obtained mean and standard deviation for each SHAPS item from three samples of healthy participants, four samples of chronic pain patients, and one MDD sample. Item-level data for other groups were not available to us at the time of writing.

Data for two healthy samples were collected in spring 2019. The study was conducted within the context of a psychology course at the University of Oslo and was approved by an internal review board. Subjects were informed that participation was voluntary and that they could withdraw from the study at any time. The samples consisted of 342 healthy Norwegian people (Sample 1: $n = 190$, 107 females, age $M \pm SD = 34.85 \pm 12.64$; Sample 2: $n = 152$, 31 females, age $M \pm SD = 24.54 \pm 5.99$) without any current major psychiatric conditions.

Data collection for the MDD sample and age- and sex-matched healthy controls is ongoing, but the data used here were collected between August 2017 and October 2019 in a Swedish-speaking sample (MDD: $n = 64$, 44 females, age $M \pm SD = 36.16 \pm 13.60$; Healthy control: $n = 34$, 23 females, age $M \pm SD = 32.68 \pm 11.05$). Treatment seeking patients were recruited through advertisements in local newspapers or referred from general practitioners or the adult psychiatric clinic at Linköping University Hospital, Sweden. Healthy controls were recruited through advertisements on social media and flyers at public buildings. Eligibility to participate was assessed by a trained interviewer using the Mini-International Neuropsychiatric Interview,[30] a validated clinical interview for diagnosis of psychiatric disorders according to Diagnostic and Statistical Manual of Mental Disorders, Fifth Edition (DSM-5) or International Statistical Classification of Diseases, 10th revision (ICD-10) criteria. Participants had to meet criteria for an ongoing MDD episode (MDD group), or no current major psychiatric disorder or a history thereof (healthy control). Exclusion criteria for the MDD group were, amongst others, any medical condition that accounts for the depression symptoms as determined by the study physician, current DSM-5 diagnosis of substance dependence (except nicotine) or current DMS-5 diagnosis of psychotic disorder (except MDD with mood congruent psychotic features). Patients had to be free of any antidepressant or psychotropic medication, with the exception of a stable dose of fluoxetine over the past one month. In addition, MDD patients who failed to respond to Escitalopram within the past year were excluded. To extensively assess psychiatric symptoms, MDD patients were further examined by a clinician using the Comprehensive Psychopathological Rating Scale.[31] Following inclusion, participants provided informed consent and were asked to complete several questionnaires, including a Swedish version of the SHAPS. The study was approved by the Regional Ethical Board of Linköping (Dnr 2017/17-31).

Details on all four samples of chronic pain patients are available in.[32]

For the healthy and the chronic pain group, we used random-effects models with the DL estimator to calculate summary mean and 95% confidence intervals (CI) for each SHAPS item. For the MDD sample, we simply calculated individual SHAPS items means and 95% CIs.

eAppendix 2. Results

2.1 Article and sample characteristics

All included articles were made available between 1999 and 2019, with 91% being published in peer-reviewed journals. These articles were almost exclusively written in English (99%). Most samples were European (45%) and North American (41%), followed by Asian (10%), Australian (2%) and South American (1%). Sample age ranged from 13.04-72.01 years. The percentage of female participants ranged from 0-100%.

2.2 Group characteristics

Group characteristics are available in eTable 8. The clinical groups were on average older than the healthy group. While schizophrenia, SUD and PD groups comprised a larger proportion of male participants compared to the healthy group, the current MDD group consisted of a greater proportion of female participants relative to healthy group. The gender distribution within these groups mirror gender differences in the actual prevalence of these disorders (i.e. MDD is more common in women than men[33], while SUD [34] and PD [35] are more common in men than women). General depression symptoms were elevated in all clinical groups. The most severe depression occurred in current MDD. Compared to the healthy group, the reported prevalence of clinically significant anhedonia according to the original SHAPS cut-off (SHAPS > 2 under 0-1 scoring) was generally higher in SCZ (*ns*), SUD, PD and chronic pain, and even higher in current MDD.

2.3 Quality assessment

Modifications other than translation occurred in only four samples (2%). For one sample, "freshly baked bread" was replaced with "freshly boiled rice" to make a single item more culturally appropriate.[36] Another sample rated their experiences "over the past week" rather than "in the last few days".[37] Finally, two samples were assessed with the SHAPS-C.[38] This version is administered by a clinician and therefore alters the wording of the items. However, it still assess the same pleasures, uses 1-4 scoring, and is highly correlated with the original SHAPS.[39] Scores obtained with these SHAPS versions would likely correspond largely with those from the original SHAPS, and the modifications did therefore not warrant exclusion of the samples.

2.4 Sensitivity analysis

Results from sensitivity analyses were fully consistent with the results obtained in the primary analyses. When substituting the DerSimonian-Laird method for the PM and REML methods, the results remained largely the same (eTable 1-4). Adding a random effect at the article level did not lead to any notable changes in the results (eTable 5-6).

2.5 Item-level group comparison

We found no evidence that the available clinical groups showed specific anhedonia to certain items. Instead, the patients with chronic pain scored uniformly higher than healthy participants and uniformly lower than the MDD group, consistent with prior reports that the SHAPS reflects one latent factor.[40]

© 2020 Trøstheim M et al. *JAMA Network Open.*

eTable 1. Effect size meta-analyses with the PM estimator of the between-studies variance

Group	k	Hedges' g [95% CI]	T	Q (df)	I^2
MDD (current)	38	2.22 [1.96, 2.47]	0.73	201.64 (37)***	85%
MDD (remitted)	5	0.07 [-0.18, 0.32]	0.00	3.86 (4)	0%
BD	5	0.39 [-0.29, 1.08]	0.74	31.97 (4)***	91%
SCZ	13	0.62 [0.45, 0.78]	0.20	19.46 (12)	46%
SUD	6	0.82 [0.61, 1.02]	0.00	1.43 (5)	0%
PD	7	0.45 [0.09, 0.81]	0.40	15.06 (6)*	75%

Note. The number of samples is denoted by *k*. MDD = major depressive disorder, BD = bipolar disorder, SCZ = schizophrenia, SUD = substance use disorders, PD = Parkinson's disease. * $p < .05$, *** $p < .0001$.

© 2020 Trøstheim M et al. *JAMA Network Open.*

eTable 2. Between-groups comparisons using meta-regression with the PM estimator of the between-studies variance

	1-4 scoring			0-1 scoring			Effect size comparisons		
**Comparison**	**B (SE)**	**z**	**p**	**B (SE)**	**z**	**p**	**B (SE)**	**z**	**p**
Healthy vs MDD (current)	12.83 (0.54)	23.60	< .0001	5.12 (0.26)	19.56	< .0001	---	---	---
Healthy vs MDD (remitted)	0.99 (0.91)	1.09	.28	---	---	---	---	---	---
MDD (remitted) vs MDD (current)	11.84 (1.30)	9.13	< .0001	---	---	---	2.12 (0.36)	5.93	< .0001
Healthy vs SCZ	3.01 (0.84)	3.59	.0003	2.07 (0.39)	5.28	< .0001	---	---	---
SCZ vs MDD (current)	9.77 (1.19)	8.23	< .0001	3.09 (0.65)	4.74	< .0001	1.61 (0.22)	7.31	< .0001
Healthy vs SUD	4.63 (0.93)	5.00	< .0001	1.21 (0.22)	5.55	< .0001	---	---	---
SUD vs MDD (current)	8.16 (1.32)	6.18	< .0001	3.92 (0.53)	7.41	< .0001	1.40 (0.32)	4.39	< .0001
Healthy vs PD	2.49 (1.18)	2.11	.03	0.83 (0.24)	3.52	.0004	---	---	---
PD vs MDD (current)	10.18 (1.67)	6.11	< .0001	4.24 (0.43)	9.86	< .0001	1.75 (0.31)	5.70	< .0001
Healthy vs Chronic pain	4.00 (0.96)	4.19	< .0001	1.00 (0.25)	3.93	< .0001	---	---	---
Chronic pain vs MDD (current)	8.81 (1.39)	6.33	< .0001	4.16 (0.66)	6.34	< .0001	---	---	---

Note. 1-4 and 0-1 scoring: *B* and *SE* are on the same scale as the SHAPS. Effect size: *B* and *SE* are on the same scale as Hedges' g. MDD = major depressive disorder, SCZ = schizophrenia, SUD = substance use disorders, PD = Parkinson's disease. "---" = not applicable.

© 2020 Trøstheim M et al. *JAMA Network Open.*

eTable 3. Effect size meta-analyses with the REML estimator of the between-studies variance

Group	k	Hedges' g [95% CI]	T	Q (df)	I^2
MDD (current)	38	2.21 [1.97, 2.46]	0.69	201.64 (37)***	84%
MDD (remitted)	5	0.07 [-0.18, 0.32]	0.00	3.86 (4)	0%
BD	5	0.39 [-0.29, 1.07]	0.73	31.97 (4)***	91%
SCZ	13	0.63 [0.48, 0.77]	0.14	19.46 (12)	27%
SUD	6	0.82 [0.61, 1.02]	0.00	1.43 (5)	0%
PD	7	0.45 [0.13, 0.76]	0.33	15.06 (6)*	67%

Note. The number of samples is denoted by *k*. MDD = major depressive disorder, BD = bipolar disorder, SCZ = schizophrenia, SUD = substance use disorders, PD = Parkinson's disease. * $p < .05$, *** $p < .0001$.

© 2020 Trøstheim M et al. *JAMA Network Open.*

eTable 4. Between-groups comparisons using meta-regression with the REML estimator of the between-studies variance

	**1-4 scoring**			**0-1 scoring**			**Effect size comparisons**		
**Comparison**	**B (SE)**	**z**	**p**	**B (SE)**	**z**	**p**	**B (SE)**	**z**	**p**
Healthy vs MDD (current)	12.83 (0.55)	23.53	< .0001	5.12 (0.25)	20.44	< .0001	---	---	---
Healthy vs MDD (remitted)	0.99 (0.91)	1.09	.28	---	---	---	---	---	---
MDD (remitted) vs MDD (current)	11.84 (1.31)	9.06	< .0001	---	---	---	2.12 (0.34)	6.25	< .0001
Healthy vs SCZ	3.01 (0.84)	3.60	.0003	2.07 (0.39)	5.38	< .0001	---	---	---
SCZ vs MDD (current)	9.77 (1.19)	8.18	< .0001	3.10 (0.67)	4.61	< .0001	1.60 (0.20)	7.85	< .0001
Healthy vs SUD	4.63 (0.93)	4.98	< .0001	1.21 (0.20)	6.01	< .0001	---	---	---
SUD vs MDD (current)	8.16 (1.33)	6.13	< .0001	3.92 (0.54)	7.29	< .0001	1.39 (0.30)	4.65	< .0001
Healthy vs PD	2.49 (1.18)	2.11	.03	0.82 (0.21)	3.89	< .0001	---	---	---
PD vs MDD (current)	10.18 (1.68)	6.07	< .0001	4.24 (0.43)	9.87	< .0001	1.75 (0.29)	6.01	< .0001
Healthy vs Chronic pain	4.00 (0.96)	4.18	< .0001	1.00 (0.23)	4.30	< .0001	---	---	---
Chronic pain vs MDD (current)	8.81 (1.40)	6.29	< .0001	4.16 (0.67)	6.21	< .0001	---	---	---

Note. 1-4 and 0-1 scoring: *B* and *SE* are on the same scale as the SHAPS. Effect size: *B* and *SE* are on the same scale as Hedges' g. MDD = major depressive disorder, SCZ = schizophrenia, SUD = substance use disorders, PD = Parkinson's disease. "---" = not applicable.

© 2020 Trøstheim M et al. *JAMA Network Open.*

eTable 5. Effect size meta-analyses with random effect at the article level

Group	k	Hedges' g [95% CI]	T	Q (df)
MDD (current)	38	2.21 [1.97, 2.46]	0.69	201.64 (37)***
MDD (remitted)	5	0.07 [-0.18, 0.32]	0.00	3.86 (4)
BD	5	0.57 [-0.14, 1.28]	0.48	31.97 (4)***
SCZ	13	0.63 [0.48, 0.77]	0.14	19.46 (12)
SUD	6	0.82 [0.61, 1.02]	0.00	1.43 (5)
PD	7	0.45 [0.13, 0.76]	0.33	15.06 (6)*

Note. The number of samples is denoted by *k*. MDD = major depressive disorder, BD = bipolar disorder, SCZ = schizophrenia, SUD = substance use disorders, PD = Parkinson's disease. For MDD (current and remitted), SCZ and PD, each level of the outer factor (article level) contained only a single level of the inner factor (sample level). * $p < .05$, *** $p < .0001$.

© 2020 Trøstheim M et al. *JAMA Network Open.*

eTable 6. Between-groups comparisons using meta-regression with random effect added at the article level

	1-4 scoring			0-1 scoring			Effect size comparisons		
**Comparison**	**B (SE)**	**z**	**p**	**B (SE)**	**z**	**p**	**B (SE)**	**z**	**p**
Healthy vs MDD (current)	12.83 (0.54)	23.54	< .0001	5.18 (0.23)	22.30	< .0001	---	---	---
Healthy vs MDD (remitted)	0.62 (0.61)	1.01	.31	---	---	---	---	---	---
MDD (remitted) vs MDD (current)	12.14 (1.30)	9.34	< .0001	---	---	---	2.21 (0.32)	6.85	< .0001
Healthy vs SCZ	3.98 (0.50)	7.99	< .0001	2.06 (0.39)	5.29	< .0001	---	---	---
SCZ vs MDD (current)	9.84 (1.20)	8.21	< .0001	3.10 (0.67)	4.61	< .0001	1.60 (0.20)	7.84	< .0001
Healthy vs SUD	4.58 (0.82)	5.58	< .0001	1.28 (0.16)	7.94	< .0001	---	---	---
SUD vs MDD (current)	8.14 (1.58)	5.15	< .0001	3.92 (0.54)	7.29	< .0001	1.41 (0.36)	3.88	.0001
Healthy vs PD	2.25 (0.89)	2.54	.01	0.87 (0.17)	5.11	< .0001	---	---	---
PD vs MDD (current)	10.18 (1.68)	6.07	< .0001	4.24 (0.43)	9.87	< .0001	1.75 (0.29)	6.01	< .0001
Healthy vs Chronic pain	3.70 (1.44)	2.57	.01	0.90 (0.32)	2.78	.0054	---	---	---
Chronic pain vs MDD (current)	9.13 (2.16)	4.22	< .0001	4.32 (1.05)	4.14	< .0001	---	---	---

Note. 1-4 and 0-1 scoring: *B* and *SE* are on the same scale as the SHAPS. Effect size: *B* and *SE* are on the same scale as Hedges' g. MDD = major depressive disorder, SCZ = schizophrenia, SUD = substance use disorders, PD = Parkinson's disease. For SCZ (0-1 scoring), SUD (0-1 scoring) and PD (1-4 scoring, 0-1 scoring and effect size), each level of the outer factor (article level) contained only a single level of the inner factor (sample level). "---" = not applicable.

© 2020 Trøstheim M et al. *JAMA Network Open.*

eTable 7. Sample details for all included groups

Study	Group	N	Female	Age	SHAPS [95% CI]	SHAPS scoring	Hedges' g [95% CI]	Anh.	Dep.	Med.	Received missing necessary data
Addington et al. (2019)[41]	Healthy	42	48%	19.10 (3.80)	0.10 [0.01, 0.19]	0-1	NA	---	3.49 (5.40)	NA	Yes
Admon & Pizzagalli (2015)[42]	Healthy	30	73%	30.20 (11.10)	20.80 [18.69, 22.91]	1-4	NA	---	1.90 (3.33)	NA	Yes
	MDD (remitted)	30	73%	31.20 (12.50)	21.10 [19.28, 22.92]	1-4	0.05 [-0.45 ,0.56]	---	3.49 (3.97)	0%	Yes
Admon et al. (2017)[43]	Healthy	43	77%	25.94 (6.27)	22.01 [20.10, 23.92]	1-4	NA	---	11.10 (6.63)	NA	Yes
	MDD (current)	46	80%	27.00 (6.87)	32.85 [31.28, 34.42]	1-4	1.82 [1.33 ,2.31]	---	57.63 (14.01)	0%	Yes
Al Aïn et al. (2013)[44]	Healthy	107	59%	23.90 (3.40)	22.60 [21.76, 23.44]	1-4	NA	---	6.21 (4.73)	NA	NA
Ang et al. (2017)[45]	Healthy	479	52%	29.70 (10.70)	21.30 [20.79, 21.81]	1-4	NA	---	17.78 (16.19)	NA	NA
Arrondo et al. (2015)[46]	Healthy	21	19%	34.33 (10.11)	23.38 [21.85, 24.91]	1-4	NA	---	6.81 (7.76)	NA	Yes
	MDD (current)	24	29%	33.08 (9.15)	33.42 [30.70, 36.14]	1-4	1.78 [1.09 ,2.47]	---	51.78 (11.21)	54%	Yes
	SCZ	21	14%	32.24 (7.44)	29.19 [26.54, 31.84]	1-4	1.13 [0.47 ,1.78]	---	33.41 (14.21)	100 %	Yes
Auerbach et al. (2017)[47]	Healthy	50	100%	13.04 (0.83)	20.02 [18.75, 21.29]	1-4	NA	---	10.41 (11.91)	NA	NA
Bakic et al. (2017)[48]	Healthy	44	64%	37.89 (12.23)	0.55 [-0.09, 1.19]	0-1	NA	---	6.17 (7.73)	NA	Yes
	MDD (current)	35	77%	43.00 (11.67)	7.31 [5.96, 8.66]	0-1	2.12 [1.56 ,2.67]	---	45.81 (13.78)	0%	Yes
Balducci (2009)[49]	SUD (current use)	40	30%	40.00 (11.21)	3.02 [2.03, 4.02]	0-1	NA	22%	26.85 (10.14)	NA	NA
Ballard et al. (2018)[50]	BD (depressed)	41	56%	46.38 (10.73)	36.90 [34.53, 39.27]	1-4	NA	---	49.00 (9.72)	0%	NA
Barch et al. (2014)[51]	Healthy	39	51%	37.40 (9.20)	17.20 [16.29, 18.11]	1-4	NA	---	4.60 (7.14)	NA	Yes
	SCZ	59	42%	39.30 (8.12)	22.80 [20.25, 25.35]	1-4	0.70 [0.28 ,1.11]	---	18.10 (18.57)	90%	Yes

© 2020 Trøstheim M et al. *JAMA Network Open.*

Study	Group	N	Female	Age	SHAPS [95% CI]	SHAPS scoring	Hedges' g [95% CI]	Anh.	Dep.	Med.	Received missing necessary data
Barra et al. (2007)[52]	MDD (current)	46	---	---	2.89 [1.89, 3.89]	0-1	NA	16%	---	---	NA
Boehm et al. (2018)[53]	Healthy	62	100%	19.11 (4.15)	0.87 [0.48, 1.26]	0-1	NA	---	7.27 (8.33)	NA	NA
Boger et al. (2014)[54]	SUD (current use)	40	40%	17.07 (0.98)	2.35 [1.42, 3.28]	0-1	NA	---	37.22 (21.48)	NA	Yes
Carpinelli et al. (2019)[55]	Chronic pain	120	57%	40.80 (14.50)	1.30 [1.03, 1.57]	0-1	NA	18%	16.51 (12.06)	NA	NA
	Chronic pain	120	57%	40.80 (14.50)	23.40 [22.90, 23.90]	1-4	NA	18%	16.51 (12.06)	NA	Yes
Chamberlain et al. (2019)[56]	Healthy	54	69%	34.20 (---)	16.20 [15.24, 17.16]	1-4	NA	---	1.91 (2.70)	NA	NA
	MDD (current)	198	68%	36.02 (---)	29.86 [28.91, 30.80]	1-4	2.18 [1.82 ,2.54]	---	32.87 (15.15)	76%	NA
Chase et al. (2017)[57]	Healthy	52	56%	21.30 (1.76)	19.02 [17.63, 20.41]	1-4	NA	---	17.02 (6.49)	NA	Yes
Chodkiewicz et al. (2017)[58]	Healthy	300	62%	23.50 (5.69)	2.03 [1.70, 2.36]	0-1	NA	---	13.36 (12.94)	NA	Yes
Chuang et al. (2014)[59]	Healthy	20	20%	34.30 (10.37)	23.30 [21.70, 24.90]	1-4	NA	---	1.30 (1.98)	NA	Yes
Chung & Barch (2015)[60]	Healthy	27	44%	35.56 (8.61)	17.30 [16.07, 18.53]	1-4	NA	---	3.40 (5.63)	NA	NA
Chung et al. (2016)[61]	PD	364	---	---	2.75 [2.45, 3.05]	0-1	NA	---	33.62 (12.17)	---	NA
Colic et al. (2019)[62]	Healthy	32	59%	33.09 (8.24)	0.37 [0.09, 0.65]	0-1	NA	---	1.10 (1.62)	NA	Yes
Coloigner et al. (2019)[63]	MDD (current)	114	62%	48.20 (15.30)	5.50 [4.77, 6.23]	0-1	NA	---	45.17 (9.83)	100 %	NA
Cooper et al. (2014)[64]	Healthy	38	47%	24.39 (4.76)	0.92 [0.44, 1.40]	0-1	NA	---	8.22 (8.27)	NA	NA
Culbreth, Gold et al. (2016)[65]	Healthy	36	47%	36.60 (9.20)	17.60 [16.59, 18.61]	1-4	NA	---	4.76 (7.62)	NA	Yes
	SCZ	57	33%	37.00 (8.60)	21.90 [19.72, 24.08]	1-4	0.62 [0.19 ,1.05]	---	16.83 (16.83)	88%	Yes

© 2020 Trøstheim M et al. *JAMA Network Open.*

Study	Group	N	Female	Age	SHAPS [95% CI]	SHAPS scoring	Hedges' g [95% CI]	Anh.	Dep.	Med.	Received missing necessary data
Culbreth, Westbrook et al. (2016)[66]	Healthy	30	53%	35.90 (8.20)	19.30 [17.19, 21.41]	1-4	NA	---	---	NA	Yes
	SCZ	33	48%	36.70 (9.25)	26.50 [22.92, 30.08]	1-4	0.82 [0.31 ,1.34]	---	---	79%	Yes
Cullen et al. (2018)[67]	MDD (current)	12	33%	16.99 (1.12)	6.50 [4.69, 8.31]	0-1	NA	---	47.27 (11.06)	---	Yes
Cunningham (2017)[68]	Healthy	47	68%	26.85 (12.36)	0.17 [0.01, 0.33]	0-1	NA	---	19.95 (8.83)	NA	NA
	MDD (current)	39	69%	31.18 (13.49)	4.87 [3.78, 5.96]	0-1	1.97 [1.45 ,2.48]	---	57.14 (13.98)	---	NA
Currie et al. (2017)[69]	Healthy	16	6%	42.90 (10.50)	0.88 [0.19, 1.56]	0-1	NA	---	1.41 (2.01)	NA	Yes
	SCZ	20	5%	44.00 (12.30)	1.70 [0.82, 2.58]	0-1	0.46 [-0.21 ,1.12]	---	18.61 (12.82)	---	Yes
De Berardis et al. (2017)[70]	MDD (current)	30	60%	27.20 (5.10)	6.60 [5.81, 7.39]	0-1	NA	---	56.40 (5.60)	0%	Yes
Dean et al. (2016)[71]	Healthy	17	53%	24.00 (4.26)	20.65 [17.95, 23.35]	1-4	NA	---	2.71 (4.98)	NA	Yes
dela Cruz et al. (2016)[72]	SUD (current use)	299	---	38.95 (10.80)	1.70 [1.46, 1.94]	0-1	NA	---	20.00 (11.48)	NA	NA
DelDonno et al. (2019)[73]	Healthy	30	53%	22.63 (3.12)	21.67 [19.39, 23.95]	1-4	NA	---	1.33 (2.25)	NA	Yes
Di Giuda et al. (2012)[74]	Healthy	17	59%	55.30 (13.70)	1.30 [0.30, 2.30]	0-1	NA	---	24.20 (13.40)	NA	Yes
	PD	21	33%	59.60 (13.40)	2.10 [1.24, 2.96]	0-1	0.38 [-0.26 ,1.03]	---	37.20 (8.00)	0%	Yes
Di Nicola et al. (2013)[75]	BD (euthymic)	107	61%	49.48 (11.66)	1.86 [1.51, 2.21]	0-1	NA	22%	3.16 (3.46)	100 %	NA
Dillon et al. (2014)[76]	Healthy	21	43%	36.62 (13.32)	0.24 [0.01, 0.47]	0-1	NA	---	10.01 (6.45)	NA	Yes
	MDD (current)	21	52%	34.33 (12.16)	5.10 [3.50, 6.70]	0-1	1.78 [1.07 ,2.49]	---	53.69 (14.17)	0%	Yes
Dillon et al. (2015)[77]	Healthy	37	62%	36.22 (14.32)	21.05 [19.32, 22.78]	1-4	NA	---	5.41 (4.81)	NA	Yes

Study	Group	N	Female	Age	SHAPS [95% CI]	SHAPS scoring	Hedges' g [95% CI]	Anh.	Dep.	Med.	Received missing necessary data
	MDD (current)	92	66%	39.16 (12.99)	33.83 [32.61, 35.05]	1-4	2.18 [1.72 ,2.65]	---	68.44 (10.63)	0%	Yes
Drijgers et al. (2012)[78]	Healthy	23	9%	65.40 (9.20)	22.00 [20.34, 23.66]	1-4	NA	---	4.00 (3.60)	NA	NA
	PD	23	9%	63.90 (9.80)	21.40 [19.76, 23.04]	1-4	-0.15 [-0.72 ,0.43]	---	11.40 (10.20)	87%	NA
Duncan et al. (2016)[79]	SCZ	28	29%	43.00 (11.50)	22.60 [20.45, 24.75]	1-4	NA	---	8.89 (9.63)	82%	Yes
Eisenstein et al. (2017)[80]	Healthy	59	56%	32.62 (9.19)	17.40 [16.41, 18.38]	1-4	NA	---	---	NA	Yes
	SCZ	65	57%	37.40 (8.30)	21.20 [19.35, 23.05]	1-4	0.62 [0.26 ,0.98]	---	---	83%	Yes
Ersche et al. (2012)[81]	Healthy	50	36%	32.80 (8.90)	0.20 [0.03, 0.37]	0-1	NA	---	3.49 (4.13)	NA	NA
	SUD (current use)	50	12%	34.30 (7.20)	1.40 [0.87, 1.93]	0-1	0.85 [0.44 ,1.25]	---	28.73 (18.89)	NA	NA
Farabaugh et al. (2015)[82]	MDD (current)	26	50%	47.19 (13.68)	4.00 [2.88, 5.12]	0-1	NA	---	36.40 (10.27)	0%	NA
Fava et al. (2018)[83]	MDD (current)	99	49%	46.16 (12.56)	7.03 [6.28, 7.78]	0-1	NA	---	41.29 (7.40)	---	Yes
Feng (2017)[84]	MDD (current)	50	60%	35.94 (10.79)	6.62 [5.69, 7.55]	0-1	NA	---	55.75 (13.17)	0%	Yes
Fervaha et al. (2013)[85]	Healthy	16	44%	27.50 (4.50)	18.50 [16.54, 20.46]	1-4	NA	---	1.11 (2.96)	NA	Yes
	SCZ	16	38%	28.00 (4.60)	19.60 [16.91, 22.29]	1-4	0.22 [-0.47 ,0.92]	---	14.81 (15.56)	100 %	Yes
Fletcher et al. (2015)[86]	MDD (current)	35	49%	44.43 (12.65)	6.54 [5.33, 7.75]	0-1	NA	---	68.01 (49.98)	71%	NA
Fortunati et al. (2015)[87]	Healthy	46	50%	38.30 (10.50)	0.52 [0.22, 0.82]	0-1	NA	---	1.14 (3.02)	NA	NA
	SCZ	53	40%	40.10 (10.50)	1.80 [1.07, 2.53]	0-1	0.60 [0.20 ,1.00]	12%	9.93 (11.70)	---	NA
Frey et al. (2015)[88]	Healthy	43	100%	19.90 (1.60)	2.52 [1.21, 3.82]	0-1	NA	---	8.96 (9.80)	NA	Yes

© 2020 Trøstheim M et al. *JAMA Network Open.*

Study	Group	N	Female	Age	SHAPS [95% CI]	SHAPS scoring	Hedges' g [95% CI]	Anh.	Dep.	Med.	Received missing necessary data
Fries et al. (2018)[89]	SUD (current use)	48	23%	45.81 (8.70)	25.68 [23.51, 27.85]	1-4	NA	13%	---	NA	NA
Gadeikis et al. (2017)[90]	Healthy	99	60%	33.99 (13.81)	18.96 [17.95, 19.98]	1-4	NA	---	8.03 (7.25)	NA	Yes
Garfield et al. (2017)[91]	Healthy	33	24%	36.00 (8.90)	20.40 [18.69, 22.11]	1-4	NA	---	15.33 (15.50)	NA	NA
	SUD (pharmacotherapy)	90	28%	36.30 (6.10)	25.60 [24.13, 27.07]	1-4	0.78 [0.37 ,1.19]	---	31.50 (21.67)	NA	NA
	SUD (abstinent)	31	29%	36.00 (6.20)	26.80 [24.20, 29.40]	1-4	1.01 [0.49 ,1.53]	---	34.33 (22.00)	NA	NA
Garland et al. (2019)[32]	Chronic pain	115	68%	48.30 (13.60)	24.40 [23.18, 25.62]	1-4	NA	22%	---	NA	NA
	Chronic pain	35	9%	32.90 (8.40)	25.10 [22.55, 27.65]	1-4	NA	12%	---	NA	NA
	Chronic pain	282	63%	52.00 (12.50)	24.60 [23.75, 25.45]	1-4	NA	80%	---	NA	NA
	Chronic pain	56	57%	67.80 (9.70)	23.90 [22.38, 25.42]	1-4	NA	8%	14.68 (9.38)	NA	NA
	Chronic pain	115	68%	48.30 (13.60)	1.60 [1.12, 2.08]	0-1	NA	22%	---	NA	NA
	Chronic pain	35	9%	32.90 (8.40)	2.30 [1.41, 3.19]	0-1	NA	12%	---	NA	NA
	Chronic pain	282	63%	52.00 (12.50)	1.80 [1.50, 2.10]	0-1	NA	80%	---	NA	NA
	Chronic pain	56	57%	67.80 (9.70)	1.30 [0.80, 1.80]	0-1	NA	8%	14.68 (9.38)	NA	NA
Gheza et al. (2019)[92]	MDD (current)	34	79%	42.68 (11.69)	7.21 [5.83, 8.59]	0-1	NA	---	46.72 (12.62)	0%	Yes
Godlewska et al. (2018)[93]	Healthy	50	56%	31.30 (9.90)	18.40 [17.22, 19.58]	1-4	NA	---	1.15 (1.83)	NA	Yes
Gradin et al. (2015)[94]	Healthy	25	68%	25.44 (5.02)	18.12 [16.79, 19.45]	1-4	NA	---	0.58 (1.17)	NA	Yes
	MDD (current)	25	68%	25.48 (5.52)	34.12 [32.34, 35.90]	1-4	3.93 [2.98 ,4.88]	---	35.09 (11.49)	0%	Yes

© 2020 Trøstheim M et al. *JAMA Network Open.*

Study	Group	N	Female	Age	SHAPS [95% CI]	SHAPS scoring	Hedges' g [95% CI]	Anh.	Dep.	Med.	Received missing necessary data
Grassi et al. (2019)[95]	Healthy	40	20%	37.90 (11.79)	0.50 [0.05, 0.95]	0-1	NA	0%	2.60 (2.88)	NA	Yes
Greenberg et al. (2015)[96]	Healthy	31	61%	38.42 (15.74)	20.52 [18.60, 22.44]	1-4	NA	---	2.69 (5.01)	NA	Yes
	MDD (current)	148	66%	37.11 (12.93)	33.46 [32.58, 34.34]	1-4	2.35 [1.90 ,2.81]	---	38.80 (8.90)	0%	Yes
Han (2017)[97]	Healthy	18	72%	30.11 (9.89)	22.61 [18.93, 26.29]	1-4	NA	---	8.62 (7.03)	NA	Yes
	MDD (remitted)	16	81%	31.75 (11.60)	20.44 [18.56, 22.31]	1-4	-0.33 [-1.01 ,0.34]	---	10.85 (6.17)	0%	Yes
Horndasch et al. (2016)[98]	Healthy	36	100%	23.91 (8.28)	19.34 [17.92, 20.76]	1-4	NA	---	7.25 (6.70)	NA	Yes
Huhn et al. (2016)[99]	Healthy	10	40%	25.10 (2.50)	0.20 [-0.05, 0.45]	0-1	NA	---	---	NA	NA
	SUD (abstinent)	36	25%	28.80 (9.70)	1.56 [0.87, 2.25]	0-1	0.71 [-0.01 ,1.43]	14%	---	NA	NA
Huneke et al. (2017)[100]	Healthy	60	50%	23.40 (4.29)	21.25 [20.01, 22.49]	1-4	NA	---	13.97 (6.47)	NA	Yes
Janiri et al. (2005)[101]	SUD (abstinent)	70	33%	33.00 (8.10)	1.60 [1.11, 2.09]	0-1	NA	13%	9.32 (8.18)	NA	NA
Janzen et al. (2019)[102]	MDD (current)	19	58%	47.80 (11.30)	4.05 [2.77, 5.34]	0-1	NA	11%	47.60 (13.13)	68%	NA
Kang et al. (2018)[103]	Healthy	28	39%	26.79 (6.91)	18.59 [17.00, 20.18]	1-4	NA	---	8.00 (2.18)	NA	NA
	MDD (current)	31	32%	29.96 (9.73)	37.06 [34.96, 39.16]	1-4	3.48 [2.67 ,4.29]	---	40.52 (6.38)	---	NA
Kaufmann (2017)[104]	Healthy	20	100%	24.00 (3.32)	0.32 [-0.04, 0.69]	0-1	NA	---	5.32 (5.34)	NA	Yes
Kirkpatrick et al. (2016)[105]	Healthy	97	70%	23.30 (4.10)	21.00 [20.16, 21.84]	1-4	NA	---	7.50 (10.00)	NA	Yes
Koch et al. (2018)[106]	MDD (current)	30	53%	29.90 (8.90)	34.10 [31.77, 36.43]	1-4	NA	---	44.31 (11.69)	100 %	Yes
Kos et al. (2017)[107]	Healthy	39	67%	22.69 (2.27)	1.08 [0.26, 1.90]	0-1	NA	---	9.86 (10.08)	NA	NA

© 2020 Trøstheim M et al. *JAMA Network Open.*

Study	Group	N	Female	Age	SHAPS [95% CI]	SHAPS scoring	Hedges' g [95% CI]	Anh.	Dep.	Med.	Received missing necessary data
Kumar et al. (2008)[108]	Healthy	18	61%	42.00 (12.80)	18.30 [16.31, 20.29]	1-4	NA	---	4.76 (4.44)	NA	Yes
	MDD (current)	15	60%	45.30 (12.30)	35.00 [31.61, 38.39]	1-4	2.95 [1.97 ,3.94]	---	41.37 (11.81)	100 %	Yes
Kumar et al. (2015)[109]	Healthy	10	80%	29.70 (10.14)	0.40 [-0.20, 1.00]	0-1	NA	---	3.02 (6.14)	NA	Yes
	MDD (current)	12	50%	35.83 (14.90)	5.42 [3.12, 7.72]	0-1	1.56 [0.61 ,2.52]	---	40.08 (14.40)	0%	Yes
Kumar et al. (2018)[110]	Healthy	25	76%	26.12 (8.06)	18.60 [16.84, 20.36]	1-4	NA	---	0.75 (1.52)	NA	Yes
	MDD (current)	22	73%	25.00 (5.53)	33.27 [31.57, 34.97]	1-4	3.35 [2.46 ,4.24]	---	38.29 (11.24)	0%	Yes
Lally et al. (2014)[111]	BD (depressed)	36	58%	46.69 (11.09)	37.19 [34.82, 39.56]	1-4	NA	31%	56.53 (8.35)	100 %	NA
Lally et al. (2015)[112]	MDD (current)	52	37%	48.29 (12.84)	37.69 [35.88, 39.50]	1-4	NA	45%	55.20 (7.97)	0%	NA
Lampe et al. (2001)[113]	Healthy	64	64%	71.50 (7.10)	0.69 [0.40, 0.98]	0-1	NA	---	---	NA	NA
Lampe et al. (2004)[114]	Healthy	60	100%	64.80 (11.50)	0.28 [0.13, 0.43]	0-1	NA	---	---	NA	NA
Lansdall et al. (2017)[115]	Healthy	50	54%	70.60 (6.50)	18.60 [17.38, 19.82]	1-4	NA	---	6.67 (6.35)	NA	NA
Lawson et al. (2017)[116]	Healthy	25	44%	27.44 (8.75)	21.80 [19.31, 24.29]	1-4	NA	---	4.62 (4.80)	NA	Yes
	MDD (current)	25	40%	27.76 (9.01)	32.96 [30.21, 35.71]	1-4	1.64 [1.00 ,2.28]	---	39.21 (10.85)	0%	Yes
Lemke & Schleidt (1999)[117]	Healthy	22	41%	38.80 (14.90)	1.30 [0.71, 1.89]	0-1	NA	---	---	NA	NA
Lemke (2002)[118]	PD	15	53%	66.87 (5.76)	6.13 [4.72, 7.54]	0-1	NA	---	46.82 (7.94)	100 %	NA
Lemke et al. (2005)[119]	Healthy	50	48%	63.30 (7.40)	1.62 [1.18, 2.06]	0-1	NA	---	---	NA	NA
	PD	626	45%	67.70 (9.20)	3.39 [3.10, 3.68]	0-1	0.50 [0.21 ,0.78]	286 %	64.54 (23.08)	100 %	NA

© 2020 Trøstheim M et al. *JAMA Network Open.*

Study	Group	N	Female	Age	SHAPS [95% CI]	SHAPS scoring	Hedges' g [95% CI]	Anh.	Dep.	Med.	Received missing necessary data
Lemke, Koethe et al. (1999)[120]	Healthy	8	62%	43.80 (12.90)	0.90 [0.14, 1.66]	0-1	NA	---	22.33 (10.67)	NA	NA
	MDD (current)	12	58%	42.40 (12.00)	5.90 [3.75, 8.05]	0-1	1.57 [0.55 ,2.59]	---	61.17 (12.63)	67%	NA
Lemke, Puhl et al. (1999)[121]	MDD (current)	52	65%	43.00 (12.50)	7.40 [6.58, 8.22]	0-1	NA	---	39.60 (12.80)	96%	NA
Lemke, Wendorff et al. (2000)[122]	Healthy	16	50%	43.60 (11.00)	0.30 [-0.09, 0.69]	0-1	NA	---	---	NA	NA
	MDD (current)	16	50%	44.10 (12.80)	4.10 [2.43, 5.77]	0-1	1.50 [0.72 ,2.28]	---	51.00 (14.00)	50%	NA
Lempert & Pizzagalli (2010)[123]	Healthy	36	56%	26.30 (7.50)	21.57 [20.24, 22.90]	1-4	NA	---	7.59 (8.11)	NA	NA
Lewandowski et al. (2016)[124]	Healthy	29	59%	31.00 (10.00)	0.14 [-0.02, 0.30]	0-1	NA	---	15.85 (9.91)	NA	Yes
	BD (psychosis)	42	55%	29.60 (8.40)	1.87 [1.08, 2.66]	0-1	0.84 [0.35 ,1.33]	---	27.30 (17.49)	---	Yes
	SCZ	37	30%	35.00 (11.90)	1.76 [0.94, 2.58]	0-1	0.82 [0.32 ,1.33]	---	26.09 (20.22)	---	Yes
Lin et al. (2018)[125]	Healthy	125	44%	33.15 (12.54)	0.35 [0.18, 0.52]	0-1	NA	---	---	NA	Yes
	BD (psychosis)	122	46%	39.81 (13.59)	1.58 [1.13, 2.03]	0-1	0.64 [0.39 ,0.90]	---	---	---	Yes
	SCZ	136	66%	44.26 (12.36)	1.93 [1.54, 2.32]	0-1	0.87 [0.61 ,1.12]	---	---	---	Yes
Liu et al. (2011)[126]	Healthy	87	52%	26.21 (6.50)	22.63 [21.47, 23.78]	1-4	NA	---	16.99 (6.37)	NA	NA
Liu et al. (2014)[127]	Healthy	27	56%	34.14 (10.16)	22.44 [19.26, 25.62]	1-4	NA	---	5.43 (10.00)	NA	NA
Liu et al. (2016)[128]	Healthy	107	56%	32.51 (9.26)	22.78 [21.76, 23.80]	1-4	NA	---	8.29 (4.81)	NA	NA
Liu et al. (2017)[129]	MDD (current)	21	57%	30.70 (8.90)	28.50 [26.40, 30.60]	1-4	NA	---	48.65 (10.00)	0%	NA
Liu, Wang, Zhao et al. (2012)[130]	Healthy	61	49%	26.11 (5.65)	20.83 [19.48, 22.18]	1-4	NA	---	4.32 (3.62)	NA	NA

© 2020 Trøstheim M et al. *JAMA Network Open.*

Study	Group	N	Female	Age	SHAPS [95% CI]	SHAPS scoring	Hedges' g [95% CI]	Anh.	Dep.	Med.	Received missing necessary data
	MDD (current)	71	48%	27.33 (8.90)	28.80 [27.19, 30.41]	1-4	1.27 [0.89 ,1.64]	---	44.24 (12.06)	---	NA
Liu, Wang, Zhu et al. (2012)[131]	Healthy	72	60%	30.88 (10.45)	21.50 [20.31, 22.69]	1-4	NA	---	4.62 (4.60)	NA	NA
	MDD (current)	141	55%	30.84 (10.41)	28.15 [26.87, 29.43]	1-4	0.95 [0.65 ,1.25]	---	32.56 (20.33)	---	NA
	SCZ	72	50%	33.12 (11.09)	24.25 [22.61, 25.89]	1-4	0.44 [0.11 ,0.77]	---	15.71 (16.16)	---	NA
Loas et al. (2009)[132]	SCZ	43	30%	42.00 (12.57)	2.28 [1.61, 2.95]	0-1	NA	---	29.83 (20.41)	---	Yes
Loas et al. (2014)[133]	PD	49	43%	64.84 (10.84)	1.24 [0.81, 1.67]	0-1	NA	9%	29.38 (14.13)	100 %	NA
Lorenzini (2015)[134]	Healthy	11	64%	39.55 (5.75)	0.18 [-0.06, 0.42]	0-1	NA	---	2.45 (3.22)	NA	Yes
	MDD (current)	23	65%	34.87 (9.74)	6.91 [5.67, 8.16]	0-1	2.59 [1.65 ,3.54]	---	45.24 (11.00)	---	Yes
Mann et al. (2013)[135]	Healthy	39	51%	36.46 (9.12)	17.23 [16.24, 18.22]	1-4	NA	---	---	NA	NA
	SCZ	54	39%	38.85 (8.13)	22.21 [19.64, 24.78]	1-4	0.65 [0.23 ,1.07]	---	---	100 %	NA
Martino et al. (2018)[136]	Healthy	1697	55%	37.10 (15.80)	1.15 [1.10, 1.20]	0-1	NA	253 %	---	NA	NA
Martinotti, Cloninger et al. (2008)[137]	SUD (abstinent)	50	34%	33.40 (9.30)	2.01 [1.29, 2.73]	0-1	NA	22%	10.45 (9.55)	NA	Yes
Martinotti, Di Nicola et al. (2008)[138]	SUD (abstinent)	102	33%	40.30 (11.80)	1.60 [1.19, 2.01]	0-1	NA	24%	---	NA	Yes
Matsui et al. (2013)[139]	PD	117	61%	69.40 (9.10)	1.00 [0.71, 1.29]	0-1	NA	18%	25.08 (15.40)	---	NA
Mazza et al. (2013)[140]	SCZ	12	42%	27.92 (8.90)	3.70 [2.82, 4.58]	0-1	NA	---	---	0%	Yes
McCabe et al. (2012)[141]	Healthy	25	64%	18.60 (1.60)	21.80 [20.17, 23.43]	1-4	NA	---	5.71 (5.40)	NA	Yes

© 2020 Trøstheim M et al. *JAMA Network Open.*

Study	Group	N	Female	Age	SHAPS [95% CI]	SHAPS scoring	Hedges' g [95% CI]	Anh.	Dep.	Med.	Received missing necessary data
Mies et al. (2019)[142]	Healthy	27	37%	15.50 (1.71)	24.00 [22.30, 25.70]	1-4	NA	---	---	NA	Yes
Milders et al. (2016)[143]	MDD (current)	17	47%	49.50 (11.80)	6.47 [4.89, 8.05]	0-1	NA	---	55.17 (18.11)	100 %	Yes
Misaki et al. (2016)[144]	Healthy	45	27%	32.00 (9.30)	18.30 [17.04, 19.56]	1-4	NA	---	4.68 (4.63)	NA	NA
	MDD (current)	44	27%	35.30 (11.10)	28.90 [27.07, 30.73]	1-4	1.97 [1.47 ,2.48]	---	36.47 (11.27)	0%	NA
Miura et al. (2012)[36]	PD	86	55%	72.01 (9.07)	1.19 [0.79, 1.59]	0-1	NA	14%	---	100 %	NA
Morris et al. (2019)[145]	Healthy	23	57%	38.50 (10.89)	17.58 [15.83, 19.33]	1-4	NA	---	0.90 (1.77)	NA	Yes
	MDD (current)	24	42%	38.48 (11.69)	36.39 [34.05, 38.73]	1-4	3.60 [2.67 ,4.52]	---	47.80 (10.48)	0%	Yes
Mrochen et al. (2016)[146]	PD	57	37%	64.10 (11.00)	0.70 [0.39, 1.01]	0-1	NA	5%	30.06 (14.41)	---	NA
Nagayama et al. (2012)[147]	Healthy	22	55%	62.90 (17.30)	0.55 [0.03, 1.07]	0-1	NA	---	---	NA	NA
	PD	48	54%	67.70 (9.00)	1.80 [0.99, 2.61]	0-1	0.50 [-0.01 ,1.01]	---	---	---	NA
Nagayama et al. (2017)[148]	Healthy	62	45%	70.30 (8.50)	0.40 [0.17, 0.63]	0-1	NA	3%	16.67 (12.54)	NA	NA
	PD	318	56%	67.90 (9.60)	0.88 [0.69, 1.07]	0-1	0.30 [0.03 ,0.57]	39%	21.75 (12.54)	---	NA
Nakonezny et al. (2010)[40]	MDD (current)	461	84%	50.20 (10.60)	4.00 [3.70, 4.30]	0-1	NA	---	48.23 (11.08)	---	NA
Nakonezny et al. (2015)[149]	MDD (current)	122	82%	47.00 (9.90)	3.10 [2.57, 3.63]	0-1	NA	---	41.53 (10.10)	100 %	NA
Ng et al. (2014)[150]	Healthy	82	43%	37.01 (12.58)	22.89 [21.44, 24.34]	1-4	NA	---	---	NA	NA
Norbury, et al. (2015)[151]	Healthy	45	62%	24.30 (3.55)	24.30 [22.83, 25.77]	1-4	NA	---	---	NA	Yes
Nord et al. (2018)[152]	Healthy	28	46%	26.79 (8.48)	0.89 [0.25, 1.53]	0-1	NA	---	4.29 (4.68)	NA	Yes

© 2020 Trøstheim M et al. *JAMA Network Open.*

Study	Group	N	Female	Age	SHAPS [95% CI]	SHAPS scoring	Hedges' g [95% CI]	Anh.	Dep.	Med.	Received missing necessary data
	MDD (current)	26	38%	27.96 (8.75)	5.62 [4.31, 6.93]	0-1	1.75 [1.12 ,2.37]	---	37.86 (11.29)	0%	Yes
Nugent et al. (2018)[153]	Healthy	26	62%	33.90 (10.40)	18.50 [16.85, 20.15]	1-4	NA	---	2.01 (2.14)	NA	Yes
	MDD (current)	35	60%	35.50 (9.60)	39.50 [38.21, 40.79]	1-4	5.09 [4.05 ,6.12]	---	48.30 (9.50)	0%	Yes
Osuch et al. (2016)[154]	Healthy	40	50%	20.05 (1.26)	21.75 [20.16, 23.34]	1-4	NA	---	7.54 (8.69)	NA	Yes
	MDD (current)	34	59%	19.69 (2.00)	30.57 [28.51, 32.63]	1-4	1.56 [1.03 ,2.08]	---	41.61 (18.38)	44%	Yes
Pechtel & Pizzagalli (2013)[155]	Healthy	16	100%	30.44 (10.78)	18.56 [16.31, 20.81]	1-4	NA	---	2.65 (2.83)	NA	Yes
	MDD (remitted)	31	100%	28.10 (8.14)	20.68 [18.88, 22.48]	1-4	0.42 [-0.19 ,1.03]	---	8.45 (8.02)	0%	Yes
Pechtel et al. (2013)[156]	Healthy	36	64%	31.75 (12.47)	21.06 [19.45, 22.67]	1-4	NA	---	2.11 (2.84)	NA	Yes
	MDD (remitted)	47	83%	27.87 (9.87)	20.74 [19.47, 22.01]	1-4	-0.07 [-0.50 ,0.37]	---	6.79 (6.10)	0%	Yes
Peciña et al. (2017)[157]	MDD (current)	26	38%	37.00 (13.80)	29.40 [25.71, 33.09]	1-4	NA	---	53.23 (14.36)	0%	Yes
Pettorruso et al. (2014)[158]	PD	154	44%	66.60 (9.70)	1.07 [0.88, 1.26]	0-1	NA	---	19.31 (13.75)	---	NA
Pizzagalli et al. (2019)[159]	Healthy	23	57%	26.49 (7.26)	20.74 [18.48, 23.00]	1-4	NA	---	0.93 (2.05)	NA	Yes
	MDD (current)	25	76%	26.52 (5.92)	33.20 [31.59, 34.81]	1-4	2.53 [1.77 ,3.29]	---	38.39 (10.66)	0%	Yes
Polimeni et al. (2010)[160]	Healthy	20	15%	28.60 (10.80)	1.50 [0.67, 2.33]	0-1	NA	---	---	NA	Yes
	SCZ	20	15%	42.00 (10.40)	0.90 [0.37, 1.43]	0-1	-0.37 [-1.00 ,0.25]	---	10.04 (8.80)	100 %	Yes
Pomponi et al. (2014)[161]	PD	24	46%	64.00 (7.75)	2.15 [0.80, 3.50]	0-1	NA	9%	33.80 (12.61)	---	NA
Pontieri et al. (2015)[162]	PD	155	31%	64.45 (8.78)	0.49 [0.33, 0.65]	0-1	NA	---	16.04 (9.99)	100 %	Yes

© 2020 Trøstheim M et al. *JAMA Network Open.*

Study	Group	N	Female	Age	SHAPS [95% CI]	SHAPS scoring	Hedges' g [95% CI]	Anh.	Dep.	Med.	Received missing necessary data
Redlich et al. (2015)[163]	Healthy	34	47%	38.59 (12.28)	0.52 [0.11, 0.93]	0-1	NA	---	2.73 (3.36)	NA	Yes
	MDD (current)	33	52%	38.48 (12.08)	6.26 [4.88, 7.64]	0-1	1.91 [1.33 ,2.49]	---	46.69 (13.29)	97%	Yes
	BD (depressed)	33	48%	38.12 (12.55)	4.35 [2.98, 5.72]	0-1	1.28 [0.75 ,1.80]	---	42.60 (11.38)	97%	Yes
Renfroe et al. (2016)[164]	Healthy	15	40%	70.00 (6.94)	20.47 [18.30, 22.64]	1-4	NA	---	4.13 (3.87)	NA	Yes
	PD	15	27%	66.00 (8.14)	21.41 [19.36, 23.46]	1-4	0.22 [-0.50 ,0.94]	---	14.29 (7.94)	---	Yes
Ricciardi et al. (2016)[38]	Healthy	20	60%	56.50 (10.80)	15.40 [14.13, 16.67]	1-4	NA	---	12.40 (15.00)	NA	NA
	PD	20	35%	61.40 (9.80)	26.80 [22.55, 31.05]	1-4	1.56 [0.85 ,2.27]	---	17.40 (11.60)	---	NA
Richards et al. (2017)[165]	MDD (current)	429	---	---	34.99 [34.76, 35.22]	1-4	NA	---	---	---	NA
Rizvi (2015)[166]	MDD (current)	15	67%	47.40 (10.30)	10.20 [8.83, 11.57]	0-1	NA	---	74.67 (10.83)	---	NA
Rothkirch et al. (2017)[167]	Healthy	30	73%	36.13 (11.96)	0.33 [0.02, 0.64]	0-1	NA	---	1.69 (2.85)	NA	NA
	MDD (current)	28	54%	36.32 (11.88)	5.60 [4.27, 6.93]	0-1	2.02 [1.39 ,2.65]	---	48.69 (10.31)	0%	NA
Ryu (2013)[168]	Healthy	24	54%	31.88 (6.69)	27.42 [24.10, 30.74]	1-4	NA	---	---	NA	Yes
	BD (euthymic)	20	60%	34.75 (4.41)	25.55 [23.31, 27.79]	1-4	-0.26 [-0.86 ,0.34]	---	4.00 (5.50)	100 %	Yes
	BD (manic)	24	54%	34.46 (9.11)	23.21 [21.41, 25.01]	1-4	-0.62 [-1.20 ,-0.04]	---	8.05 (4.93)	100 %	Yes
Rzepa & McCabe (2016)[169]	Healthy	18	67%	16.33 (1.60)	20.77 [17.07, 24.47]	1-4	NA	---	3.62 (6.56)	NA	Yes
Santangelo, Morgante et al. (2009,	Healthy	74	43%	---	0.86 [0.57, 1.15]	0-1	NA	---	---	NA	NA

© 2020 Trøstheim M et al. *JAMA Network Open.*

Study	Group	N	Female	Age	SHAPS [95% CI]	SHAPS scoring	Hedges' g [95% CI]	Anh.	Dep.	Med.	Received missing necessary data
Validation study)[170]											
Santangelo, Morgante et al. (2009, Observational study)[170]	PD	939	41%	67.00 (9.40)	1.80 [1.67, 1.93]	0-1	NA	---	---	---	NA
Santangelo, Vitale et al. (2009)[171]	PD	125	41%	64.00 (9.93)	1.36 [1.11, 1.61]	0-1	NA	---	22.98 (10.75)	---	NA
Scheidegger et al. (2012)[172]	Healthy	19	53%	40.50 (7.50)	20.00 [18.10, 21.90]	1-4	NA	---	---	NA	Yes
Schneier et al. (2018)[173]	Healthy	24	50%	26.90 (5.50)	18.80 [16.84, 20.76]	1-4	NA	---	7.61 (5.89)	NA	Yes
	MDD (current)	23	48%	26.70 (6.50)	32.00 [29.22, 34.78]	1-4	2.20 [1.47 ,2.92]	---	54.81 (13.28)	0%	Yes
Sobesky (2017)[174]	Healthy	18	100%	21.61 (3.18)	0.12 [-0.04, 0.28]	0-1	NA	---	1.19 (1.78)	NA	Yes
	MDD (current)	13	92%	23.00 (4.38)	4.30 [1.96, 6.64]	0-1	1.47 [0.66 ,2.27]	---	39.84 (9.71)	0%	Yes
Spalletta et al. (2013)[175]	PD	254	34%	64.30 (10.70)	0.50 [0.38, 0.62]	0-1	NA	12%	16.83 (11.75)	83%	NA
Spalletta et al. (2014)[176]	PD	24	29%	63.50 (9.30)	0.04 [-0.04, 0.12]	0-1	NA	---	12.83 (7.76)	0%	Yes
Sprengelmeyer et al. (2011)[177]	Healthy	21	57%	42.00 (12.90)	18.30 [16.46, 20.14]	1-4	NA	---	4.92 (4.60)	NA	NA
	MDD (current)	17	53%	45.60 (12.30)	35.00 [31.82, 38.18]	1-4	2.97 [2.05 ,3.90]	---	41.37 (10.81)	100 %	NA
Steele et al. (2007)[178]	Healthy	14	50%	43.00 (13.30)	18.10 [15.69, 20.51]	1-4	NA	---	1.75 (3.02)	NA	NA
Stevens et al. (2007)[179]	Healthy	26	0%	28.60 (6.50)	19.80 [18.03, 21.57]	1-4	NA	---	7.62 (2.38)	NA	NA
	SUD (current use)	25	0%	30.40 (5.70)	24.30 [22.30, 26.30]	1-4	0.91 [0.34 ,1.49]	---	34.13 (19.37)	NA	NA

© 2020 Trøstheim M et al. *JAMA Network Open.*

Study	Group	N	Female	Age	SHAPS [95% CI]	SHAPS scoring	Hedges' g [95% CI]	Anh.	Dep.	Med.	Received missing necessary data
	SUD (abstinent)	26	0%	32.50 (5.00)	22.30 [20.84, 23.76]	1-4	0.58 [0.03 ,1.14]	---	17.62 (10.16)	NA	NA
Stroud et al. (2018)[180]	MDD (current)	17	35%	44.94 (11.51)	6.82 [4.91, 8.73]	0-1	NA	---	69.93 (8.26)	41%	Yes
Szczepanik et al. (2017)[181]	Healthy	23	43%	31.80 (8.00)	19.60 [17.56, 21.64]	1-4	NA	---	2.70 (4.44)	NA	NA
	MDD (current)	21	38%	35.50 (7.50)	37.80 [35.40, 40.20]	1-4	3.38 [2.46 ,4.30]	---	51.90 (15.08)	0%	NA
Taalman (2017)[182]	Healthy	9	56%	46.78 (12.96)	0.22 [-0.07, 0.51]	0-1	NA	---	5.08 (7.94)	NA	NA
Taubitz (2015)[183]	MDD (remitted)	47	21%	22.09 (4.79)	22.89 [21.26, 24.52]	1-4	NA	---	31.98 (15.66)	28%	NA
Tonioni et al. (2014)[184]	Healthy	38	5%	27.20 (10.40)	3.10 [1.32, 4.88]	0-1	NA	---	2.20 (2.80)	NA	Yes
Tremblay et al. (2002)[185]	Healthy	36	33%	31.83 (11.02)	0.71 [0.27, 1.15]	0-1	NA	---	2.37 (3.67)	NA	Yes
	MDD (current)	40	68%	44.15 (10.67)	5.10 [3.95, 6.25]	0-1	1.52 [1.01 ,2.03]	---	44.70 (12.02)	0%	Yes
Tremblay et al. (2005)[186]	Healthy	12	58%	29.33 (9.31)	0.08 [-0.09, 0.25]	0-1	NA	---	0.72 (1.55)	NA	NA
	MDD (current)	12	50%	34.83 (13.96)	6.00 [3.44, 8.56]	0-1	1.78 [0.84 ,2.73]	---	48.65 (9.93)	0%	NA
Tudge et al. (2015)[187]	Healthy	20	50%	25.40 (4.50)	17.80 [15.74, 19.86]	1-4	NA	---	2.16 (3.63)	NA	Yes
Ubl et al. (2015)[188]	Healthy	28	54%	43.96 (12.85)	20.71 [19.13, 22.29]	1-4	NA	---	2.66 (3.86)	NA	Yes
Versace et al. (2019)[189]	Healthy	49	45%	47.00 (11.32)	22.45 [20.92, 23.98]	1-4	NA	---	12.00 (8.50)	NA	Yes
Vidotto et al. (2014)[190]	SCZ	13	54%	24.20 (2.80)	7.60 [7.17, 8.03]	0-1	NA	---	---	---	Yes
Vrieze, Ceccarini et al. (2013)[191]	Healthy	10	100%	33.30 (8.20)	17.90 [16.09, 19.71]	1-4	NA	---	2.70 (2.49)	NA	Yes
	Healthy	63	60%	44.50 (11.60)	0.40 [0.18, 0.62]	0-1	NA	---	---	NA	Yes

© 2020 Trøstheim M et al. *JAMA Network Open.*

Study	Group	N	Female	Age	SHAPS [95% CI]	SHAPS scoring	Hedges' g [95% CI]	Anh.	Dep.	Med.	Received missing necessary data
Vrieze, Pizzagalli et al. (2013)[192]	MDD (current)	79	61%	45.00 (11.90)	7.30 [6.51, 8.09]	0-1	2.49 [2.05 ,2.93]	---	33.80 (9.80)	96%	Yes
Walsh, Browning et al. (2018)[193]	Healthy	42	69%	30.21 (8.13)	20.02 [18.69, 21.35]	1-4	NA	---	12.22 (7.08)	NA	Yes
	MDD (current)	46	72%	29.52 (9.01)	34.09 [32.56, 35.62]	1-4	2.85 [2.26 ,3.44]	---	52.41 (12.05)	---	Yes
Walsh, Huneke et al. (2018)[194]	Healthy	40	50%	23.82 (4.12)	22.00 [20.37, 23.63]	1-4	NA	---	13.99 (6.89)	NA	Yes
Wardle et al. (2017)[195]	SUD (current use)	85	18%	45.53 (8.54)	25.00 [23.15, 26.85]	1-4	NA	26%	---	NA	NA
Witt et al. (2008)[196]	PD	123	37%	59.79 (7.70)	0.80 [0.54, 1.06]	0-1	NA	---	16.26 (8.78)	---	NA
Yang et al. (2017)[197]	Healthy	28	46%	28.61 (6.92)	21.75 [19.51, 23.99]	1-4	NA	---	12.63 (8.67)	NA	NA
Yoshida et al. (2017)[198]	Healthy	65	43%	34.80 (13.00)	23.30 [21.79, 24.81]	1-4	NA	---	10.98 (9.37)	NA	NA
	MDD (current)	58	57%	42.80 (11.90)	37.80 [36.38, 39.22]	1-4	2.45 [1.98 ,2.92]	---	49.05 (14.29)	---	NA
Young et al. (2013)[199]	Healthy	32	69%	34.40 (10.45)	18.25 [16.03, 20.47]	1-4	NA	---	0.74 (2.39)	NA	Yes
	MDD (current)	16	69%	38.10 (10.40)	29.30 [27.24, 31.36]	1-4	1.88 [1.17 ,2.59]	---	37.18 (11.99)	0%	Yes
Young et al. (2017)[200]	MDD (current)	36	72%	31.53 (10.69)	31.77 [30.02, 33.53]	1-4	NA	---	40.21 (15.45)	0%	Yes
Young, Bellgowan et al. (2014)[201]	Healthy	16	62%	27.30 (8.02)	19.10 [15.84, 22.36]	1-4	NA	---	0.91 (2.01)	NA	Yes
	MDD (current)	16	62%	34.20 (9.06)	29.90 [26.66, 33.14]	1-4	1.59 [0.79 ,2.38]	---	41.50 (15.15)	0%	Yes
	MDD (remitted)	16	62%	31.60 (12.30)	21.50 [19.02, 23.98]	1-4	0.40 [-0.30 ,1.09]	---	6.55 (8.70)	0%	Yes
Young, Zotev et al. (2014)[37]	MDD (current)	21	86%	37.33 (9.70)	30.33 [27.68, 32.99]	1-4	NA	---	45.01 (10.84)	0%	Yes
Yuan et al. (2018)[202]	Healthy	20	0%	34.00 (9.00)	23.50 [20.96, 26.04]	1-4	NA	---	4.35 (6.87)	NA	Yes

© 2020 Trøstheim M et al. *JAMA Network Open.*

Study	Group	N	Female	Age	SHAPS [95% CI]	SHAPS scoring	Hedges' g [95% CI]	Anh.	Dep.	Med.	Received missing necessary data
Zahodne et al. (2012)[203]	PD	95	32%	66.24 (9.94)	23.04 [21.69, 24.39]	1-4	NA	---	15.66 (8.78)	---	Yes

Note. MDD = major depressive disorder, BD = bipolar disorder, SCZ = schizophrenia, SUD = substance use disorders, PD = Parkinson's disease. Except for N, numbers indicate percentage, mean (SD) or mean [95% CI]. Anh. = Anhedonic. Anhedonic is the percent of participants scoring above the original SHAPS cut-off (> 2 under 0-1 scoring). Dep. = Depression. Depression score is standardized (possible range 0-100). Med. = Medicated. Medicated indicates the percentage of patients ON medications at the time of assessment. NA = not applicable, "---" = data not available.

© 2020 Trøstheim M et al. *JAMA Network Open.*

eTable 8. Group characteristics

Group	k	N	Female	Age	Depression	Medicated	Anhedonic
Healthy	113	6541	55% (0-100%)[b]	33.95 ± 11.34 (13.04-71.50)[b]	8.74 ± 8.99 (0.58-24.2)[b]	---	14% (0-15%)[b]
MDD (current)	61	3350	64% (27-92%)[a]	39.50 ± 11.08 (16.99-50.20)[a]	45.33 ± 13.40 (32.56-74.67)[a]	38% (0-100%)	62% (35-87%)[a]
MDD (remitted)	6	187	67% (21-100%)[a]	27.64 ± 9.50 (22.09-31.76)[a,b]	13.19 ± 9.72 (3.49-31.98)[a,b]	7% (0-28%)	---
BD	8	425	54% (46-61%)[b]	41.78 ± 11.58 (29.60-49-48)[a,b]	23.79 ± 9.25 (3.16-56.53)[a,b]	84% (0-100%)	37% (21-86%)[a,b]
SCZ	17	739	44% (5-66%)[a,b]	38.56 ± 10.28 (24.20-44.26)[a]	18.06 ± 16.17 (8.89-33.41)[a,b]	87% (0-100%)	23% (---)[b]
SUD	14	992	26% (0-40%)[a,b]	37.13 ± 9.26 (17.07-45.81)[a,b]	22.70 ± 14.80 (9.32-37.22)[a,b]	---	31% (19-55%)[a,b]
PD	22	3652	43% (9-61%)[a,b]	66.46 ± 9.49 (59.60-72.01)[a,b]	33.02 ± 5.42 (11.40-64.54)[a,b]	93% (0-100%)	25% (5-46%)[a,b]
Chronic pain	5	608	59% (9-68%)[b]	49.45 ± 12.72 (32.90-67.80)[a,b]	15.93 ± 11.28 (14.68-16.51)[a,b]	---	23% (14-34%)[a,b]

Note. Percent or M ± SD. Range in parenthesis. The number of samples is denoted by *k*. Depression score is standardized (possible range 0-100). MDD = major depressive disorder, BD = bipolar disorder, SCZ = schizophrenia, SUD = substance use disorders, PD = Parkinson's disease. Depression score is standardized (possible range 0-100). Medicated indicates the percentage of patients ON medications at the time of assessment. Anhedonic is the percent of participants scoring above the original SHAPS cut-off (> 2 under 0-1 scoring). "---" = not applicable. [a] Different from healthy, $p < .05$. [b] Different from MDD (current), $p < .05$.

© 2020 Trøstheim M et al. *JAMA Network Open.*

eTable 9. Sample details for the anorexia nervosa, obsessive-compulsive disorder and posttraumatic stress disorder groups

**Study**	**Group**	**N**	**Female**	**Age**	**SHAPS [95% CI]**	**SHAPS scoring**	**Hedges' g [95% CI]**	**Anh.**	**Dep.**	**Med.**	**Received missing necessary data**
Boehm et al. (2018)[53]	AN	35	100%	15.78 (2.56)	2.43 [1.59, 3.27]	0-1	0.78 [0.35, 1.21]	---	29.40 (19.87)	100%	NA
Kaufmann (2017)[104]	AN	16	100%	22.69 (4.57)	3.19 [3.02, 3.36]	0-1	4.20 [3.03, 5.37]	---	32.75 (15.56)	38%	Yes
Abramovitch et al. (2014)[204]	OCD	113	63%	35.75 (12.55)	1.92 [1.85, 1.99]	0-1	NA	28%	64.19 (54.05)	---	NA
Grassi et al. (2019)[95]	OCD	44	7%	34.68 (12.17)	2.80 [1.94, 3.66]	0-1	0.98 [0.53, 1.43]	18%	9.78 (5.40)	93%	Yes
Olson et al. (2018)[205]	PTSD	21	57%	34.11 (6.94)	30.90 [28.10, 33.70]	1-4	NA	---	32.22 (17.90)	14%	NA
Vidotto et al. (2014)[190]	PTSD	25	56%	35.20 (14.10)	8.38 [7.07, 9.69]	0-1	NA	---	---	---	Yes
Yuan et al. (2018)[202]	PTSD	36	0%	32.00 (7.00)	29.90 [28.04, 31.76]	1-4	1.10 [0.52, 1.68	---	28.63 (12.43)	0%	Yes

Note. These samples met the inclusion criteria, but the number of samples within each group was insufficient for inclusion in the statistical analyses. AN = anorexia nervosa, OCD = obsessive-compulsive disorder, PTSD = posttraumatic stress disorder. Except for N, numbers indicate percentage, mean (SD) or mean [95% CI]. Anh. = Anhedonic. Anhedonic is the percent of participants scoring above the original SHAPS cut-off (> 2 under 0-1 scoring). Dep. = Depression. Depression score is standardized (possible range 0-100). Med. = Medicated. Medicated indicates the percentage of patients ON medications at the time of assessment. NA = not applicable, "---" = data not available.

© 2020 Trøstheim M et al. *JAMA Network Open.*

eTable 10. Completeness of necessary data for each included group

	Healthy	MDD		BD	SCZ	SUD	PD	Chronic pain
		**Current**	**Past**					
Total samples	150	80	8	11	22	20	30	5
Included	113 (75%)	61 (76%)	6 (75%)	8 (73%)	17 (77%)	14 (70%)	22 (73%)	5 (100%)
Available data	45 (30%)	25 (31%)	1 (13%)	3 (27%)	3 (14%)	11 (55%)	17 (57%)	4 (80%)
Received data	68 (45%)	36 (45%)	5 (63%)	5 (45%)	14 (64%)	3 (15%)	5 (17%)	1 (20%)
Still missing data	37 (25%)	19 (24%)	2 (25%)	3 (27%)	5 (22%)	6 (30%)	8 (27%)	0 (0%)

Note. Necessary data included the number of participants, SHAPS mean and standard deviation, and SHAPS scoring method. All numbers indicate *k* samples and percentage of total samples. 'Included' indicates the number of samples included for each group. 'Available data' indicates the number of samples for which necessary data was available in the article. 'Received data' indicates the number of samples for which we received necessary data. 'Still missing data' indicates samples for which necessary data is still missing despite repeated emails to study authors. MDD = major depressive disorder, BD = bipolar disorder, SCZ = schizophrenia, SUD = substance use disorders, PD = Parkinson's disease.

© 2020 Trøstheim M et al. *JAMA Network Open.*

eTable 11. Reporting of comorbidity for clinical samples

	MDD		BD	SCZ	SUD	PD	Chronic pain
	**Current**	**Past**					
Total samples	61 (100%)	6 (100%)	8 (100%)	17 (100%)	14 (100%)	22 (100%)	5 (100%)
**Comorbid major depression**							
No comorbidity	---	---	3 (38%)	6 (35%)	9 (64%)	6 (27%)	0 (0%)
At least some comorbidity	---	---	3 (38%)	0 (0%)	3 (21%)	5 (23%)	3 (60%)
Not reported	---	---	2 (25%)	11 (65%)	2 (14%)	11 (50%)	2 (40%)
**Comorbid psychotic symptoms or disorders**							
No comorbidity	51 (84%)	4 (67%)	6 (75%)	---	12 (86%)	14 (64%)	3 (60%)
At least some comorbidity	2 (3%)	1 (17%)	2 (25%)	---	0 (0%)	0 (0%)	0 (0%)
Not reported	8 (13%)	1 (17%)	0 (0%)	---	2 (14%)	8 (36%)	2 (40%)
**Comorbid substance dependence, abuse or disorders**							
No comorbidity	48 (79%)	4 (67%)	7 (88%)	14 (82%)	---	8 (36%)	1 (20%)
At least some comorbidity	2 (3%)	1 (17%)	0 (0%)	0 (0%)	---	0 (0%)	3 (60%)
Not reported	11 (18%)	1 (17%)	1 (12%)	3 (18%)	---	14 (64%)	1 (20%)
**Comorbid anxiety disorders**							
No comorbidity	11 (18%)	1 (17%)	2 (25%)	2 (12%)	6 (43%)	5 (23%)	0 (0%)
At least some comorbidity	12 (20%)	3 (50%)	1 (13%)	0 (0%)	2 (14%)	2 (9%)	0 (0%)
Not reported	38 (62%)	2 (33%)	5 (63%)	15 (88%)	6 (43%)	15 (68%)	5 (100%)

Note. All numbers indicate *k* samples and percentage of total samples. 'No comorbidity' was defined as no participants with comorbid symptoms/disorders. 'At least some comorbidity' was defined as ≥ 1 participant(s) with comorbid symptoms/disorders. MDD = major depressive disorder, BD = bipolar disorder, SCZ = schizophrenia, SUD = substance use disorders, PD = Parkinson's disease. "---" = not applicable.

© 2020 Trøstheim M et al. *JAMA Network Open.*

eTable 12. Between-groups comparisons adjusting for age

Comparison			Group			Age			
**1-4 scoring**	**Model**	**k**	**B (SE)**	**z**	**p**	**B (SE)**	**z**	**p**	**R^2**
Healthy vs MDD (current)	1	102	12.75 (0.56)	22.83	< .0001	---	---	---	84%
Healthy vs MDD (current)	2	102	12.76 (0.55)	23.02	< .0001	-0.02 (0.03)	-0.67	.50	84%
Healthy vs SCZ	1	81	3.01 (0.85)	3.55	.0004	---	---	---	8%
Healthy vs SCZ	2	81	3.18 (0.82)	3.86	.0001	-0.05 (0.02)	-1.93	.0540	16%
Healthy vs SUD	1	78	4.64 (0.96)	4.81	< .0001	---	---	---	18%
Healthy vs SUD	2	78	4.85 (0.94)	5.17	< .0001	-0.04 (0.02)	-1.79	.07	25%
Healthy vs PD	1	76	2.50 (1.21)	2.06	.04	---	---	---	3%
Healthy vs PD	2	76	4.00 (1.40)	2.86	.0042	-0.05 (0.02)	-1.96	.0498	12%
Healthy vs Chronic pain	1	77	4.00 (0.97)	4.11	< .0001	---	---	---	30%
Healthy vs Chronic pain	2	77	4.71 (1.01)	4.66	< .0001	-0.04 (0.02)	-1.94	.0520	35%
**0-1 scoring**	**Model**	**k**	**B (SE)**	**z**	**p**	**B (SE)**	**z**	**p**	**R^2**
Healthy vs MDD (current)	1	69	5.24 (0.19)	27.57	< .0001	---	---	---	70%
Healthy vs MDD (current)	2	69	5.25 (0.19)	27.15	< .0001	-0.00 (0.01)	-0.53	.60	69%
Healthy vs SCZ	1	48	2.14 (0.29)	7.47	< .0001	---	---	---	28%
Healthy vs SCZ	2	48	2.13 (0.29)	7.33	< .0001	-0.01 (0.01)	-1.63	.10	24%
Healthy vs SUD	1	48	1.21 (0.23)	5.22	< .0001	---	---	---	14%
Healthy vs SUD	2	48	1.21 (0.23)	5.18	< .0001	-0.00 (0.01)	-0.14	.89	14%
Healthy vs PD	1	57	0.73 (0.20)	3.71	.0002	---	---	---	0%
Healthy vs PD	2	57	0.73 (0.29)	2.50	.0125	0.00 (0.01)	0.02	.99	0%
Healthy vs Chronic pain	1	45	1.00 (0.27)	3.67	.0002	---	---	---	9%
Healthy vs Chronic pain	2	45	1.02 (0.28)	3.61	.0003	-0.00 (0.01)	-0.27	.78	9%
**Effect size**	**Model**	**k**	**Intercept (SE)**	**z**	**p**	**B (SE)**	**z**	**p**	**R^2**
Healthy vs MDD (current)	1	38	2.21 (0.12)	18.59	< .0001	---	---	---	---
Healthy vs MDD (current)	2	38	2.25 (0.14)	16.18	< .0001	-0.02 (0.04)	-0.65	.52	0%
Healthy vs SCZ	1	13	0.62 (0.08)	7.72	< .0001	---	---	---	---
Healthy vs SCZ	2	13	0.69 (0.11)	6.02	< .0001	-0.02 (0.02)	-0.99	.32	0%
Healthy vs SUD	1	6	0.82 (0.10)	7.81	< .0001	---	---	---	---
Healthy vs SUD	2	6	0.92 (0.16)	5.90	< .0001	-0.07 (0.08)	-0.87	.38	---
Healthy vs PD	1	7	0.44 (0.15)	3.02	.0025	---	---	---	---
Healthy vs PD	2	7	0.34 (0.15)	2.26	.02	0.07 (0.04)	1.77	.08	18%

Note. 1-4 and 0-1 scoring: *B* and *SE* are on the same scale as the SHAPS. Effect size: *Intercept*, *B* and *SE* are on the same scale as Hedges' g. R^2 is the amount of explained heterogeneity. The number of samples is denoted by *k*. Model 1 is without age and model 2 is with age. MDD = major depressive disorder, SCZ = schizophrenia, SUD = substance use disorders, PD = Parkinson's disease. "---" = not applicable.

© 2020 Trøstheim M et al. *JAMA Network Open.*

eTable 13. Between-groups comparisons adjusting for percent female participants

Comparison			Group			% Female			
**1-4 scoring**	**Model**	**k**	**B (SE)**	**z**	**p**	**B (SE)**	**z**	**p**	**R^2**
Healthy vs MDD (current)	1	102	12.75 (0.56)	22.83	< .0001	---	---	---	84%
Healthy vs MDD (current)	2	102	12.84 (0.56)	23.13	< .0001	-0.03 (0.01)	-2.17	.03	84%
Healthy vs SCZ	1	81	3.01 (0.85)	3.55	.0004	---	---	---	8%
Healthy vs SCZ	2	81	2.54 (0.86)	2.93	.0033	-0.03 (0.01)	-2.33	.02	11%
Healthy vs SUD	1	78	4.64 (0.96)	4.81	< .0001	---	---	---	17%
Healthy vs SUD	2	78	3.69 (1.09)	3.38	.0007	-0.02 (0.01)	-1.82	.07	19%
Healthy vs PD	1	76	2.50 (1.21)	2.06	.04	---	---	---	3%
Healthy vs PD	2	76	1.69 (1.27)	1.33	.18	-0.03 (0.01)	-1.95	.0511	5%
Healthy vs Chronic pain	1	77	4.00 (0.97)	4.11	< .0001	---	---	---	30%
Healthy vs Chronic pain	2	77	3.97 (0.96)	4.12	< .0001	-0.03 (0.01)	-2.17	.03	31%
**0-1 scoring**	**Model**	**k**	**B (SE)**	**z**	**p**	**B (SE)**	**z**	**p**	**R^2**
Healthy vs MDD (current)	1	70	5.23 (0.19)	27.77	< .0001	---	---	---	69%
Healthy vs MDD (current)	2	70	5.27 (0.19)	27.66	< .0001	-0.01 (0.00)	-1.73	.08	69%
Healthy vs SCZ	1	49	2.13 (0.28)	7.52	< .0001	---	---	---	28%
Healthy vs SCZ	2	49	2.18 (0.30)	7.16	< .0001	0.00 (0.00)	0.49	.63	25%
Healthy vs SUD	1	48	1.23 (0.25)	4.92	< .0001	---	---	---	9%
Healthy vs SUD	2	48	1.14 (0.28)	4.11	< .0001	-0.00 (0.00)	-0.83	.41	6%
Healthy vs PD	1	58	0.73 (0.20)	3.71	.0002	---	---	---	0%
Healthy vs PD	2	58	0.71 (0.21)	3.43	.0006	-0.00 (0.00)	-0.22	.83	0%
Healthy vs Chronic pain	1	46	0.99 (0.27)	3.67	.0002	---	---	---	9%
Healthy vs Chronic pain	2	46	0.98 (0.27)	3.58	.0003	-0.00 (0.00)	-1.20	.23	7%
**Effect size**	**Model**	**k**	**Intercept (SE)**	**z**	**p**	**B (SE)**	**z**	**p**	**R^2**
Healthy vs MDD (current)	1	38	2.21 (0.12)	18.59	< .0001	---	---	---	---
Healthy vs MDD (current)	2	38	2.21 (0.12)	18.33	< .0001	-0.01 (0.01)	-0.62	.54	0%
Healthy vs SCZ	1	13	0.62 (0.08)	7.72	< .0001	---	---	---	---
Healthy vs SCZ	2	13	0.63 (0.09)	7.13	< .0001	0.00 (0.01)	0.36	.72	0%
Healthy vs SUD	1	6	0.82 (0.10)	7.81	< .0001	---	---	---	---
Healthy vs SUD	2	6	0.82 (0.12)	7.06	< .0001	0.00 (0.01)	0.06	.96	---
Healthy vs PD	1	7	0.44 (0.15)	3.02	.0025	---	---	---	---
Healthy vs PD	2	7	0.35 (0.15)	2.34	.02	-0.02 (0.01)	-1.5	.12	13%

Note. 1-4 and 0-1 scoring: *B* and *SE* are on the same scale as the SHAPS. Effect size: *Intercept*, *B* and *SE* are on the same scale as Hedges' g. R^2 is the amount of explained heterogeneity. The number of samples is denoted by *k*. Model 1 is without percent female and model 2 is with percent female. MDD = major depressive disorder, SCZ = schizophrenia, SUD = substance use disorders, PD = Parkinson's disease. "---" = not applicable.

© 2020 Trøstheim M et al. *JAMA Network Open.*

eTable 14. Between-groups comparisons adjusting for depression severity

Comparison			Group			Depression severity			
**1-4 scoring**	**Model**	**k**	**B (SE)**	**z**	**p**	**B (SE)**	**z**	**p**	**R^2**
Healthy vs SCZ	1	70	3.28 (0.96)	3.40	.0007	---	---	---	9%
Healthy vs SCZ	2	70	1.46 (1.08)	1.35	.18	0.16 (0.05)	3.22	.0013	21%
Healthy vs SUD	1	68	4.53 (1.10)	4.11	< .0001	---	---	---	15%
Healthy vs SUD	2	68	1.40 (1.55)	0.90	.37	0.14 (0.05)	2.73	.0063	26%
Healthy vs PD	1	68	2.60 (1.14)	2.27	.02	---	---	---	3%
Healthy vs PD	2	68	1.48 (1.17)	1.26	.21	0.14 (0.05)	2.52	.0119	15%
Healthy vs Chronic pain	1	66	3.53 (1.42)	2.48	.0130	---	---	---	20%
Healthy vs Chronic pain	2	66	2.32 (1.44)	1.61	.11	0.13 (0.05)	2.38	.02	29%
**0-1 scoring**	**Model**	**k**	**B (SE)**	**z**	**p**	**B (SE)**	**z**	**p**	**R^2**
Healthy vs SCZ	1	34	1.12 (0.23)	4.84	< .0001	---	---	---	26%
Healthy vs SCZ	2	34	0.87 (0.27)	3.26	.0011	0.02 (0.01)	2.04	.04	23%
Healthy vs SUD	1	35	1.34 (0.20)	6.79	< .0001	---	---	---	55%
Healthy vs SUD	2	35	1.11 (0.25)	4.52	< .0001	0.02 (0.01)	1.66	.10	53%
Healthy vs PD	1	44	0.82 (0.21)	3.92	< .0001	---	---	---	1%
Healthy vs PD	2	44	-0.31 (0.22)	-1.44	.15	0.06 (0.01)	7.10	< .0001	51%
Healthy vs Chronic pain	1	31	0.79 (0.28)	2.84	.0046	---	---	---	27%
Healthy vs Chronic pain	2	31	0.62 (0.30)	2.06	.04	0.02 (0.01)	1.51	.13	22%
**Effect size**	**Model**	**k**	**Intercept (SE)**	**z**	**p**	**B (SE)**	**z**	**p**	**R^2**
Healthy vs SCZ	1	8	0.61 (0.08)	7.24	< .0001	---	---	---	---
Healthy vs SCZ	2	8	0.30 (0.27)	1.14	.25	0.02 (0.02)	1.20	.23	---
Healthy vs SUD	1	5	0.83 (0.11)	7.57	< .0001	---	---	---	---
Healthy vs SUD	2	5	0.53 (0.39)	1.36	.18	0.01 (0.02)	0.78	.44	---
Healthy vs PD	1	5	0.43 (0.24)	1.81	.07	---	---	---	---
Healthy vs PD	2	5	0.96 (0.46)	2.11	.04	-0.05 (0.03)	-1.39	.17	0%

Note. 1-4 and 0-1 scoring: *B* and *SE* are on the same scale as the SHAPS. Effect size: *Intercept*, *B* and *SE* are on the same scale as Hedges' g. R^2 is the amount of explained heterogeneity. The number of samples is denoted by *k*. Model 1 is without depression severity and model 2 is with depression severity. MDD = major depressive disorder, SCZ = schizophrenia, SUD = substance use disorders, PD = Parkinson's disease. "---" = not applicable.

© 2020 Trøstheim M et al. *JAMA Network Open.*

eTable15. The contribution of age to SHAPS scores

	1-4 scoring					0-1 scoring				
**Group**	**k**	**B (SE)**	**z**	**p**	**R^2**	**k**	**B (SE)**	**z**	**p**	**R^2**
Healthy	72	-0.05 (0.02)	-1.91	.06	9%	40	0.00 (0.01)	-0.10	.92	0%
MDD (current)	30	0.16 (0.09)	1.84	< .0001	9%	29	0.00 (0.04)	-0.12	.91	10%
MDD (remitted)	6	-0.20 (0.11)	-1.86	.06	100%	---	---	---	---	---
SCZ	9	-0.06 (0.22)	-0.26	.80	0%	8	-0.24 (0.06)	-3.88	.0001	80%
SUD	6	0.11 (0.11)	0.99	.32	11%	8	-0.01 (0.02)	-0.45	.65	0%
PD	4	-0.63 (0.59)	-1.06	.29	0%	17	0.07 (0.06)	1.17	.24	26%
Chronic pain	5	0.01 (0.04)	0.24	.81	0%	5	-0.01 (0.02)	-0.75	.45	0%

Note. 1-4 and 0-1 scoring: *B* and *SE* are on the same scale as the SHAPS. R^2 is the amount of explained heterogeneity. The number of samples is denoted by *k*. MDD = major depressive disorder, SCZ = schizophrenia, SUD = substance use disorders, PD = Parkinson's disease. "---" = not applicable.

© 2020 Trøstheim M et al. *JAMA Network Open.*

eTable 16. The contribution of percent female participants to SHAPS scores

	1-4 scoring					0-1 scoring				
**Group**	**k**	**B (SE)**	**z**	**p**	**R^2**	**k**	**B (SE)**	**z**	**p**	**R^2**
Healthy	72	-0.03 (0.01)	-2.08	.04	3%	41	0.00 (0.00)	-0.98	< .001	0%
MDD (current)	30	-0.03 (0.04)	-0.86	.39	0%	29	-0.02 (0.02)	-0.89	.38	31%
MDD (remitted)	6	-0.03 (0.01)	-2.29	.02	100%	---	---	---	---	---
SCZ	9	-0.10 (0.07)	-1.36	.17	4%	8	0.05 (0.05)	0.96	.34	0%
SUD	6	0.11 (0.03)	3.49	< .001	100%	7	0.02 (0.02)	1.24	.22	0%
PD	4	0.10 (0.08)	1.32	.19	6%	17	0.04 (0.02)	2.17	.03	26%
Chronic pain	5	-0.01 (0.03)	-0.35	.72	0%	5	-0.01 (0.01)	-1.10	.27	0%

Note. 1-4 and 0-1 scoring: *B* and *SE* are on the same scale as the SHAPS. R^2 is the amount of explained heterogeneity. The number of samples is denoted by *k*. MDD = major depressive disorder, SCZ = schizophrenia, SUD = substance use disorders, PD = Parkinson’s disease. “---” = not applicable.

© 2020 Trøstheim M et al. *JAMA Network Open.*

eTable 17. The contribution of percent medicated patients to SHAPS scores

	1-4 scoring					0-1 scoring					Effect size				
**Group**	**k**	**B (SE)**	**z**	**p**	**R^2**	**k**	**B (SE)**	**z**	**p**	**R^2**	**k**	**B (SE)**	**z**	**p**	**R^2**
MDD (current)	25	0.00 (0.02)	0.22	.83	1%	23	0.00 (0.01)	0.06	.95	0%	31	-0.00 (0.00)	-0.04	.97	0%
SCZ	8	0.01 (0.13)	0.10	.92	0%	---	---	---	---	---	8	-0.02 (0.02)	-0.99	.32	0%
PD	---	---	---	---	---	8	0.01 (0.01)	1.24	.21	0%	3	-0.00 (0.01)	-0.13	.89	0%

Note. 1-4 and 0-1 scoring: *B* and *SE* are on the same scale as the SHAPS. Effect size: *B* and *SE* are on the same scale as Hedges' g. R^2 is the amount of explained heterogeneity. The number of samples is denoted by *k*. MDD = major depressive disorder, SCZ = schizophrenia, PD = Parkinson's disease. "---" = not applicable

© 2020 Trøstheim M et al. *JAMA Network Open.*

eReferences

1. Trøstheim M. *Anhedonia in clinical and non-clinical populations - An exploratory meta-analysis of studies using the Snaith-Hamilton Pleasure Scale* [Master thesis], University of Oslo; 2019.
2. Trøstheim M, Eikemo M, Meir R, Hansen I, Leknes S. Anhedonia in clinical and non-clinical populations – an exploratory meta-analysis of studies using the Snaith-Hamilton Pleasure Scale. Paper presented at: European Behavioural Pharmacology Society Biannual Meeting2019; Braga, Portugal.
3. Trøstheim M, Eikemo M, Leknes S. Anhedonia in substance use disorder – results from a meta-analysis of studies using the Snaith-Hamilton Pleasure Scale. Paper presented at: Vettreseminaret2019; Lillestrøm, Norway.
4. Beck AT, Steer RA, Brown GK. *Manual for Beck Depression lnventory-II.* San Antonio, TX: Psychological Corporation; 1996.
5. Beck AT, Ward CH, Mendelson M, Mock J, Erbaugh J. An Inventory for Measuring Depression. *Archives of General Psychiatry.* 1961;4(6):561-571. https://doi.org/10.1001/archpsyc.1961.01710120031004
6. Radloff LS. The CES-D Scale: A Self-Report Depression Scale for Research in the General Population. *Applied Psychological Measurement.* 1977;1(3):385-401. https://doi.org/10.1177/014662167700100306
7. Lovibond SH, Lovibond PF. *Manual for the Depression Anxiety Stress Scales.* 2nd ed. Sydney: Psychology Foundation; 1995.
8. Hamilton M. A rating scale for depression. *Journal of neurology, neurosurgery, and psychiatry.* 1960;23(1):56-62. https://doi.org/10.1136/jnnp.23.1.56
9. Montgomery SA, Åsberg M. A New Depression Scale Designed to be Sensitive to Change. *British Journal of Psychiatry.* 1979;134(4):382-389. https://doi.org/10.1192/bjp.134.4.382
10. Costello EJ, Angold A. Scales to Assess Child and Adolescent Depression: Checklists, Screens, and Nets. *Journal of the American Academy of Child & Adolescent Psychiatry.* 1988;27(6):726-737. https://doi.org/10.1097/00004583-198811000-00011
11. Zigmond AS, Snaith RP. The Hospital Anxiety and Depression Scale. *Acta Psychiatrica Scandinavica.* 1983;67(6):361-370. https://doi.org/10.1111/j.1600-0447.1983.tb09716.x
12. Rush Aj, Giles DE, Schlesser MA, Fulton CL, Weissenburger J, Burns C. The inventory for depressive symptomatology (IDS): Preliminary findings. *Psychiatry Research.* 1986;18(1):65-87. https://doi.org/10.1016/0165-1781(86)90060-0
13. Rush AJ, Trivedi MH, Ibrahim HM, et al. The 16-Item quick inventory of depressive symptomatology (QIDS), clinician rating (QIDS-C), and self-report (QIDS-SR): a psychometric evaluation in patients with chronic major depression. *Biological Psychiatry.* 2003;54(5):573-583. https://doi.org/10.1016/S0006-3223(02)01866-8
14. Bech P, Rafaelsen OJ. The use of rating scales exemplified by a comparison of the Hamilton and the Bech-Rafaelsen Melancholia Scale. *Acta Psychiatrica Scandinavica.* 1980;62(S285):128-132. https://doi.org/10.1111/j.1600-0447.1980.tb07683.x
15. Watson D, Weber K, Assenheimer JS, Clark LA, Strauss ME, McCormick RA. Testing a tripartite model: I. Evaluating the convergent and discriminant validity of anxiety and depression symptom scales. *Journal of Abnormal Psychology.* 1995;104(1):3-14. https://doi.org/10.1037/0021-843X.104.1.3
16. Yesavage JA, Brink TL, Rose TL, et al. Development and validation of a geriatric depression screening scale: A preliminary report. *Journal of Psychiatric Research.* 1982;17(1):37-49. https://doi.org/10.1016/0022-3956(82)90033-4
17. Zung WWK. A Self-Rating Depression Scale. *Archives of General Psychiatry.* 1965;12(1):63-70. https://doi.org/10.1001/archpsyc.1965.01720310065008
18. Rockliff BW. A Brief Self-Rating Questionnaire for Depression (SRQ-D). *Psychosomatics.* 1969;10(4):236-243. https://doi.org/10.1016/S0033-3182(69)71734-0
19. Rabey JM, Bass H, Bonuccelli U, et al. Evaluation of the Short Parkinson's Evaluation Scale: a new friendly scale for the evaluation of Parkinson's disease in clinical drug trials. *Clin Neuropharmacol.* 1997;20(4):322-337. http://europepmc.org/abstract/MED/9260730.
20. Addington D, Addington J, Maticka-tyndale E. Assessing Depression in Schizophrenia: The Calgary Depression Scale. *British Journal of Psychiatry.* 1993;163(Supplement 22):39-44. https://doi.org/10.1192/S0007125000292581
21. Snaith RP, Hamilton M, Morley S, Humayan A, Hargreaves D, Trigwell P. A Scale for the Assessment of Hedonic Tone the Snaith–Hamilton Pleasure Scale. *British Journal of Psychiatry.* 1995;167(1):99-103. https://doi.org/10.1192/bjp.167.1.99

© 2020 Trøstheim M et al. *JAMA Network Open.*

22. The Cochrane Collaboration. Cochrane Handbook for Systematic Reviews of Interventions. In: Higgins JPT, Green S, eds.2011: https://training.cochrane.org/handbook.
23. Trøstheim M, Eikemo M, Hansen I, Alnes S, Leknes S. Anhedonia in clinical and non-clinical populations: an exploratory meta-analysis of studies using the Snaith-Hamilton Pleasure Scale (SHAPS). *PROSPERO.* 2018:CRD42018109910. http://www.crd.york.ac.uk/PROSPERO/display_record.php?ID=CRD42018109910.
24. Bero L, Chartres N, Diong J, et al. The risk of bias in observational studies of exposures (ROBINS-E) tool: concerns arising from application to observational studies of exposures. *Systematic Reviews.* 2018;7(1):242. https://doi.org/10.1186/s13643-018-0915-2
25. Borenstein M, Hedges LV, Higgins JPT, Rothstein HR. *Introduction to meta-analysis.* Chichester, UK: John Wiley & Sons; 2009.
26. Higgins JPT, Thompson SG, Deeks JJ, Altman DG. Measuring inconsistency in meta-analyses. *BMJ.* 2003;327:557-560. https://doi.org/10.1136/bmj.327.7414.557
27. López-López JA, Marín-Martínez F, Sánchez-Meca J, Van den Noortgate W, Viechtbauer W. Estimation of the predictive power of the model in mixed-effects meta-regression: A simulation study. *British Journal of Mathematical and Statistical Psychology.* 2014;67(1):30-48. https://doi.org/10.1111/bmsp.12002
28. Veroniki AA, Jackson D, Viechtbauer W, et al. Methods to estimate the between-study variance and its uncertainty in meta-analysis. *Research Synthesis Methods.* 2016;7(1):55-79. https://doi.org/10.1002/jrsm.1164
29. Konstantopoulos S. Fixed effects and variance components estimation in three-level meta-analysis. *Research Synthesis Methods.* 2011;2(1):61-76. https://doi.org/10.1002/jrsm.35
30. Sheehan DV, Lecrubier Y, Sheehan KH, et al. The Mini-International Neuropsychiatric Interview (M.I.N.I): The development and validation of a structured diagnostic psychiatric interview for DSM-IV and ICD-10. *The Journal of Clinical Psychiatry.* 1998;59(Suppl 20):22-33.
31. Åsberg M, Montgomery SA, Perris C, Schalling D, Sedvall G. A comprehensive psychopathological rating scale. *Acta Psychiatrica Scandinavica.* 1978;57(S271):5-27. https://doi.org/10.1111/j.1600-0447.1978.tb02357.x
32. Garland EL, Trøstheim M, Eikemo M, Ernst G, Leknes S. Anhedonia in chronic pain and prescription opioid misuse. *Psychological Medicine.* 2019:1-12. https://doi.org/10.1017/S0033291719002010
33. Nolen-Hoeksema S. Gender Differences in Depression. *Current Directions in Psychological Science.* 2001;10(5):173-176. https://doi.org/10.1111/1467-8721.00142
34. McHugh RK, Votaw VR, Sugarman DE, Greenfield SF. Sex and gender differences in substance use disorders. *Clinical Psychology Review.* 2018;66:12-23. https://doi.org/10.1016/j.cpr.2017.10.012
35. Gillies GE, Pienaar IS, Vohra S, Qamhawi Z. Sex differences in Parkinson's disease. *Front Neuroendocrinol.* 2014;35(3):370-384. https://doi.org/10.1016/j.yfrne.2014.02.002
36. Miura S, Kida H, Nakajima J, et al. Anhedonia in Japanese patients with Parkinson's disease: Analysis using the Snaith–Hamilton Pleasure Scale. *Clinical Neurology and Neurosurgery.* 2012;114(4):352-355. https://doi.org/10.1016/j.clineuro.2011.11.008
37. Young KD, Zotev V, Phillips R, et al. Real-Time fMRI Neurofeedback Training of Amygdala Activity in Patients with Major Depressive Disorder. *PLOS ONE.* 2014;9(2):e88785. https://doi.org/10.1371/journal.pone.0088785
38. Ricciardi L, Ferrazzano G, Demartini B, et al. Know thyself: Exploring interoceptive sensitivity in Parkinson's disease. *Journal of the Neurological Sciences.* 2016;364:110-115. https://doi.org/10.1016/j.jns.2016.03.019
39. Ameli R, Luckenbaugh DA, Gould NF, et al. SHAPS-C: the Snaith-Hamilton pleasure scale modified for clinician administration. *PeerJ.* 2014;2:e429. https://doi.org/10.7717/peerj.429
40. Nakonezny PA, Carmody TJ, Morris DW, Kurian BT, Trivedi MH. Psychometric evaluation of the Snaith–Hamilton Pleasure Scale (SHAPS) in adult outpatients with major depressive disorder. *International Clinical Psychopharmacology.* 2010;25(6):328-333. https://doi.org/10.1097/YIC.0b013e32833eb5ee
41. Addington J, Liu L, Goldstein BI, et al. Clinical staging for youth at-risk for serious mental illness. *Early Intervention in Psychiatry.* 2019. https://doi.org/10.1111/eip.12786
42. Admon R, Pizzagalli DA. Corticostriatal pathways contribute to the natural time course of positive mood. *Nature Communications.* 2015;6:10065. https://doi.org/10.1038/ncomms10065
43. Admon R, Kaiser RH, Dillon DG, et al. Dopaminergic Enhancement of Striatal Response to Reward in Major Depression. *American Journal of Psychiatry.* 2017;174(4):378-386. https://doi.org/10.1176/appi.ajp.2016.16010111

© 2020 Trøstheim M et al. *JAMA Network Open.*

44. Al Aïn S, Carré A, Fantini-Hauwel C, Baudouin J-Y, Besche-Richard C. What is the emotional core of the multidimensional Machiavellian personality trait? *Frontiers in Psychology.* 2013;4:454. https://doi.org/10.3389/fpsyg.2013.00454
45. Ang Y-S, Lockwood P, Apps MAJ, Muhammed K, Husain M. Distinct Subtypes of Apathy Revealed by the Apathy Motivation Index. *PLOS ONE.* 2017;12(1):e0169938. https://doi.org/10.1371/journal.pone.0169938
46. Arrondo G, Segarra N, Metastasio A, et al. Reduction in ventral striatal activity when anticipating a reward in depression and schizophrenia: a replicated cross-diagnostic finding. *Frontiers in Psychology.* 2015;6:1280. https://doi.org/10.3389/fpsyg.2015.01280
47. Auerbach RP, Pisoni A, Bondy E, et al. Neuroanatomical Prediction of Anhedonia in Adolescents. *Neuropsychopharmacology.* 2017;42:2087-2095. https://doi.org/10.1038/npp.2017.28
48. Bakic J, Pourtois G, Jepma M, Duprat R, De Raedt R, Baeken C. Spared internal but impaired external reward prediction error signals in major depressive disorder during reinforcement learning. *Depression and Anxiety.* 2017;34(1):89-96. https://doi.org/10.1002/da.22576
49. Balducci XL. *Probing Mesocorticolimbic Dopamine Function in Alcohol Dependence Using Dextroamphetamine: Behavioural and FMRI Studies* [Doctoral thesis], University of Toronto; 2009.
50. Ballard ED, Yarrington JS, Farmer CA, et al. Parsing the heterogeneity of depression: An exploratory factor analysis across commonly used depression rating scales. *Journal of Affective Disorders.* 2018;231:51-57. https://doi.org/10.1016/j.jad.2018.01.027
51. Barch DM, Treadway MT, Schoen N. Effort, anhedonia, and function in schizophrenia: Reduced effort allocation predicts amotivation and functional impairment. *Journal of Abnormal Psychology.* 2014;123(2):387-397. https://doi.org/10.1037/a0036299
52. Barra A, Camardese G, Tonioni F, et al. Plasma magnesium level and psychomotor retardation in major depressed patients. *Magnesium research.* 2007;20(4):245-249. https://doi.org/10.1684/mrh.2007.0115
53. Boehm I, Flohr L, Steding J, et al. The Trajectory of Anhedonic and Depressive Symptoms in Anorexia Nervosa: A Longitudinal and Cross-Sectional Approach. *European Eating Disorders Review.* 2018;26(1):69-74. https://doi.org/10.1002/erv.2565
54. Boger KD, Auerbach RP, Pechtel P, Busch AB, Greenfield SF, Pizzagalli DA. Co-occurring depressive and substance use disorders in adolescents: An examination of reward responsiveness during treatment. *Journal of Psychotherapy Integration.* 2014;24(2):109-121. https://doi.org/10.1037/a0036975
55. Carpinelli L, Bucci C, Santonicola A, Zingone F, Ciacci C, Iovino P. Anhedonia in irritable bowel syndrome and in inflammatory bowel diseases and its relationship with abdominal pain. *Neurogastroenterology and Motility.* 2019;31(3). https://doi.org/10.1111/nmo.13531
56. Chamberlain SR, Cavanagh J, de Boer P, et al. Treatment-resistant depression and peripheral C-reactive protein. *British Journal of Psychiatry.* 2019;214(1):11-19. https://doi.org/10.1192/bjp.2018.66
57. Chase HW, Fournier JC, Bertocci MA, et al. A pathway linking reward circuitry, impulsive sensation-seeking and risky decision-making in young adults: identifying neural markers for new interventions. *Translational Psychiatry.* 2017;7:e1096. https://doi.org/10.1038/tp.2017.60
58. Chodkiewicz J, Miniszewska J, Strzelczyk D, Gąsior K. Polish adaptation of the Psychache Scale by Ronald Holden and co-workers. *Psychiatria Polska.* 2017;51(2):369-381. https://doi.org/10.12740/PP/OnlineFirst/59448
59. Chuang J-Y, Murray GK, Metastasio A, et al. Brain structural signatures of negative symptoms in depression and schizophrenia. *Frontiers in Psychiatry.* 2014;5:116. https://doi.org/10.3389/fpsyt.2014.00116
60. Chung YS, Barch D. Anhedonia is associated with reduced incentive cue related activation in the basal ganglia. *Cognitive, Affective, & Behavioral Neuroscience.* 2015;15(4):749-767. https://doi.org/10.3758/s13415-015-0366-3
61. Chung SJ, Asgharnejad M, Bauer L, Ramirez F, Jeon B. Evaluation of rotigotine transdermal patch for the treatment of depressive symptoms in patients with Parkinson's disease. *Expert Opinion on Pharmacotherapy.* 2016;17(11):1453-1461. https://doi.org/10.1080/14656566.2016.1202917
62. Colic L, von Düring F, Denzel D, et al. Rostral Anterior Cingulate Glutamine/Glutamate Disbalance in Major Depressive Disorder Depends on Symptom Severity. *Biological Psychiatry: Cognitive Neuroscience and Neuroimaging.* 2019. https://doi.org/10.1016/j.bpsc.2019.04.003
63. Cologner J, Batail J-M, Commowick O, et al. White matter abnormalities in depression: A categorical and phenotypic diffusion MRI study. *Neuroimage-Clinical.* 2019;22. https://doi.org/10.1016/j.nicl.2019.101710

64. Cooper AJ, Duke É, Pickering AD, Smillie LD. Individual differences in reward prediction error: contrasting relations between feedback-related negativity and trait measures of reward sensitivity, impulsivity and extraversion. *Frontiers in Human Neuroscience.* 2014;8:248. https://doi.org/10.3389/fnhum.2014.00248
65. Culbreth AJ, Gold JM, Cools R, Barch DM. Impaired Activation in Cognitive Control Regions Predicts Reversal Learning in Schizophrenia. *Schizophrenia Bulletin.* 2016;42(2):484-493. https://doi.org/10.1093/schbul/sbv075
66. Culbreth AJ, Westbrook A, Daw ND, Botvinick M, Barch DM. Reduced model-based decision-making in schizophrenia. *Journal of Abnormal Psychology.* 2016;125(6):777-787. https://doi.org/10.1037/abn0000164
67. Cullen KR, Amatya P, Roback MG, et al. Intravenous Ketamine for Adolescents with Treatment-Resistant Depression: An Open-Label Study. *Journal of Child and Adolescent Psychopharmacology.* 2018;28(7):437-444. https://doi.org/10.1089/cap.2018.0030
68. Cunningham S. *Childhood Maltreatment and Mechanisms of Vulnerability Within Anhedonia and Depression* [Master thesis], Queen's University; 2017.
69. Currie J, Buruju D, Perrin JS, Reid IC, Steele JD, Feltovich N. Schizophrenia illness severity is associated with reduced loss aversion. *Brain Research.* 2017;1664:9-16. https://doi.org/10.1016/j.brainres.2017.03.006
70. De Berardis D, Fornaro M, Orsolini L, et al. Effect of agomelatine treatment on C-reactive protein levels in patients with major depressive disorder: an exploratory study in "real-world," everyday clinical practice. *CNS Spectrums.* 2017;22(4):342-347. https://doi.org/10.1017/S1092852916000572
71. Dean Z, Horndasch S, Giannopoulos P, McCabe C. Enhanced neural response to anticipation, effort and consummation of reward and aversion during bupropion treatment. *Psychological Medicine.* 2016;46(11):2263-2274. https://doi.org/10.1017/S003329171600088X
72. dela Cruz AM, Carmody T, Greer TL, et al. Baseline medical comorbidities in adults randomized in the STRIDE trial for psychostimulant use disorders. *The American Journal on Addictions.* 2016;25(3):215-220. https://doi.org/10.1111/ajad.12363
73. DelDonno SR, Karstens AJ, Cerny B, et al. The Titrated Monetary Incentive Delay Task: Sensitivity, convergent and divergent validity, and neural correlates in an RDoC sample. *Journal of Clinical and Experimental Neuropsychology.* 2019;41(5):512-529. https://doi.org/10.1080/13803395.2019.1585519
74. Di Giuda D, Camardese G, Bentivoglio AR, et al. Dopaminergic dysfunction and psychiatric symptoms in movement disorders: a 123 I-FP-CIT SPECT study. *European journal of nuclear medicine and molecular imaging.* 2012;39(12):1937-1948. https://doi.org/10.1007/s00259-012-2232-7
75. Di Nicola M, De Risio L, Battaglia C, et al. Reduced hedonic capacity in euthymic bipolar subjects: A trait-like feature? *Journal of Affective Disorders.* 2013;147(1-3):446-450. https://doi.org/10.1016/j.jad.2012.10.004
76. Dillon DG, Dobbins IG, Pizzagalli DA. Weak reward source memory in depression reflects blunted activation of VTA/SN and parahippocampus. *Social Cognitive and Affective Neuroscience.* 2014;9(10):1576-1583. https://doi.org/10.1093/scan/nst155
77. Dillon DG, Wiecki T, Pechtel P, et al. A computational analysis of flanker interference in depression. *Psychological Medicine.* 2015;45(11):2333-2344. https://doi.org/10.1017/S0033291715000276
78. Drijgers RL, Verhey FRJ, Tissingh G, van Domburg PHMF, Aalten P, Leentjens AFG. The role of the dopaminergic system in mood, motivation and cognition in Parkinson's disease: A double blind randomized placebo-controlled experimental challenge with pramipexole and methylphenidate. *Journal of the Neurological Sciences.* 2012;320(1-2):121-126. https://doi.org/10.1016/j.jns.2012.07.015
79. Duncan MJ, Faulkner G, Remington G, Arbour-Nicitopoulos K. Characterizing the affective responses to an acute bout of moderate-intensity exercise among outpatients with schizophrenia. *Psychiatry Research.* 2016;237:264-270. https://doi.org/10.1016/j.psychres.2016.01.030
80. Eisenstein SA, Bogdan R, Chen L, et al. Preliminary evidence that negative symptom severity relates to multilocus genetic profile for dopamine signaling capacity and D2 receptor binding in healthy controls and in schizophrenia. *Journal of Psychiatric Research.* 2017;86:9-17. https://doi.org/10.1016/j.jpsychires.2016.11.007
81. Ersche KD, Turton AJ, Chamberlain SR, Müller U, Bullmore ET, Robbins TW. Cognitive Dysfunction and Anxious-Impulsive Personality Traits Are Endophenotypes for Drug Dependence. *American Journal of Psychiatry.* 2012;169(9):926-936. https://doi.org/10.1176/appi.ajp.2012.11091421

© 2020 Trøstheim M et al. *JAMA Network Open.*

82. Farabaugh A, Fisher L, Nyer M, et al. Similar changes in cognitions following cognitive-behavioral therapy or escitalopram for major depressive disorder: Implications for mechanisms of change. *Annals of Clinical Psychiatry.* 2015;27(2):118-126.
83. Fava M, Freeman MP, Flynn M, et al. Double-blind, placebo-controlled, dose-ranging trial of intravenous ketamine as adjunctive therapy in treatment-resistant depression (TRD). *Molecular Psychiatry.* 2018. https://doi.org/10.1038/s41380-018-0256-5
84. Feng S. *Association between Reward Sensitivity and Smoking Status in Major Depressive Disorder* [Master thesis], Virginia Polytechnic Institute and State University; 2017.
85. Fervaha G, Graff-Guerrero A, Zakzanis KK, Foussias G, Agid O, Remington G. Incentive motivation deficits in schizophrenia reflect effort computation impairments during cost-benefit decision-making. *Journal of Psychiatric Research.* 2013;47(11):1590-1596. https://doi.org/10.1016/j.jpsychires.2013.08.003
86. Fletcher K, Parker G, Paterson A, Fava M, Iosifescu D, Pizzagalli DA. Anhedonia in melancholic and non-melancholic depressive disorders. *Journal of Affective Disorders.* 2015;184:81-88. https://doi.org/10.1016/j.jad.2015.05.028
87. Fortunati R, Ossola P, Camerlengo A, et al. Anhedonia in schizophrenia: The role of subjective experiences. *Comprehensive Psychiatry.* 2015;62:152-160. https://doi.org/10.1016/j.comppsych.2015.07.011
88. Frey A-L, Malinowska L, Harley K, et al. Investigating subtypes of reward processing deficits as trait markers for depression. *Translational Developmental Psychiatry.* 2015;3(1):27517. https://doi.org/10.3402/tdp.v3.27517
89. Fries GR, Khan S, Stamatovich S, et al. Anhedonia in cocaine use disorder is associated with inflammatory gene expression. *Plos One.* 2018;13(11). https://doi.org/10.1371/journal.pone.0207231
90. Gadeikis D, Bos N, Schweizer S, Murphy F, Dunn B. Engaging in an experiential processing mode increases positive emotional response during recall of pleasant autobiographical memories. *Behaviour Research and Therapy.* 2017;92:68-76. https://doi.org/10.1016/j.brat.2017.02.005
91. Garfield JBB, Cotton SM, Allen NB, et al. Evidence that anhedonia is a symptom of opioid dependence associated with recent use. *Drug and Alcohol Dependence.* 2017;177:29-38. https://doi.org/10.1016/j.drugalcdep.2017.03.012
92. Gheza D, Bakic J, Baeken C, De Raedt R, Pourtois G. Abnormal approach-related motivation but spared reinforcement learning in MDD: Evidence from fronto-midline Theta oscillations and frontal Alpha asymmetry. *Cognitive, Affective and Behavioral Neuroscience.* 2019. https://doi.org/10.3758/s13415-019-00693-4
93. Godlewska BR, Masaki C, Sharpley AL, Cowen PJ, Emir UE. Brain glutamate in medication-free depressed patients: a proton MRS study at 7 Tesla. *Psychological Medicine.* 2018;48(10):1731-1737. https://doi.org/10.1017/S0033291717003373
94. Gradin VB, Pérez A, MacFarlane JA, et al. Abnormal brain responses to social fairness in depression: an fMRI study using the Ultimatum Game. *Psychological Medicine.* 2015;45(6):1241-1251. https://doi.org/10.1017/S0033291714002347
95. Grassi G, Makris N, Pallanti S. Addicted to compulsion: Assessing three core dimensions of addiction across obsessive-compulsive disorder and gambling disorder. *CNS Spectrums.* 2019. https://doi.org/10.1017/S1092852919000993
96. Greenberg T, Chase HW, Almeida JR, et al. Moderation of the Relationship Between Reward Expectancy and Prediction Error-Related Ventral Striatal Reactivity by Anhedonia in Unmedicated Major Depressive Disorder: Findings From the EMBARC Study. *American Journal of Psychiatry.* 2015;172(9):881-891. https://doi.org/10.1176/appi.ajp.2015.14050594
97. Han S. *Measuring and modifying information bias in depression* [Master thesis], University of Oxford; 2017.
98. Horndasch S, O'Keefe S, Lamond A, Brown K, McCabe C. Increased anticipatory but decreased consummatory brain responses to food in sisters of anorexia nervosa patients. *BJPsych Open.* 2016;2(4):255-261. https://doi.org/10.1192/bjpo.bp.115.002550
99. Huhn AS, Meyer RE, Harris JD, et al. Evidence of anhedonia and differential reward processing in prefrontal cortex among post-withdrawal patients with prescription opiate dependence. *Brain Research Bulletin.* 2016;123:102-109. https://doi.org/10.1016/j.brainresbull.2015.12.004
100. Huneke NTM, Walsh AEL, Brown R, Browning M, Harmer CJ. No evidence for an acute placebo effect on emotional processing in healthy volunteers. *Journal of Psychopharmacology.* 2017;31(12):1578-1587. https://doi.org/10.1177/0269881117739552

© 2020 Trøstheim M et al. *JAMA Network Open.*

101. Janiri L, Martinotti G, Dario T, et al. Anhedonia and Substance-Related Symptoms in Detoxified Substance-Dependent Subjects: A Correlation Study. *Neuropsychobiology.* 2005;52(1):37-44. https://doi.org/10.1159/000086176
102. Janzen TB, Al Shirawi MI, Rotzinger S, Kennedyz SH, Bartel L. A Pilot Study Investigating the Effect of Music-Based Intervention on Depression and Anhedonia. *Frontiers in Psychology.* 2019;10. https://doi.org/10.3389/fpsyg.2019.01038
103. Kang L, Zhang A, Sun N, et al. Functional connectivity between the thalamus and the primary somatosensory cortex in major depressive disorder: a resting-state fMRI study. *Bmc Psychiatry.* 2018;18. https://doi.org/10.1186/s12888-018-1913-6
104. Kaufmann L-K. *"Reshaping" the brain – longitudinal investigation of structural and functional brain alterations during weight gain in anorexia nervosa* [Doctoral thesis], University of Fribourg; 2017.
105. Kirkpatrick MG, Goldenson NI, Kapadia N, et al. Emotional traits predict individual differences in amphetamine-induced positive mood in healthy volunteers. *Psychopharmacology.* 2016;233(1):89-97. https://doi.org/10.1007/s00213-015-4091-y
106. Koch K, Stegmaier S, Schwarz L, et al. Neural correlates of processing emotional prosody in unipolar depression. *Human Brain Mapping.* 2018;39(8):3419-3427. https://doi.org/10.1002/hbm.24185
107. Kos C, Klaasen NG, Marsman J-BC, et al. Neural basis of self-initiative in relation to apathy in a student sample. *Scientific Reports.* 2017;7:3264. https://doi.org/10.1038/s41598-017-03564-5
108. Kumar P, Waiter G, Ahearn T, Milders M, Reid I, Steele JD. Abnormal temporal difference reward-learning signals in major depression. *Brain.* 2008;131(8):2084-2093. https://doi.org/10.1093/brain/awn136
109. Kumar P, Slavich GM, Berghorst LH, et al. Perceived life stress exposure modulates reward-related medial prefrontal cortex responses to acute stress in depression. *Journal of Affective Disorders.* 2015;180:104-111. https://doi.org/10.1016/j.jad.2015.03.035
110. Kumar P, Goer F, Murray L, et al. Impaired reward prediction error encoding and striatal-midbrain connectivity in depression. *Neuropsychopharmacology.* 2018;43(7):1581-1588. https://doi.org/10.1038/s41386-018-0032-x
111. Lally N, Nugent AC, Luckenbaugh DA, Ameli R, Roiser JP, Zarate CA. Anti-anhedonic effect of ketamine and its neural correlates in treatment-resistant bipolar depression. *Translational Psychiatry.* 2014;4:e469. https://doi.org/10.1038/tp.2014.105
112. Lally N, Nugent AC, Luckenbaugh DA, Niciu MJ, Roiser JP, Zarate Jr CA. Neural correlates of change in major depressive disorder anhedonia following open-label ketamine. *Journal of Psychopharmacology.* 2015;29(5):596-607. https://doi.org/10.1177/0269881114568041
113. Lampe IK, Kahn RS, Heeren TJ. Apathy, Anhedonia, and Psychomotor Retardation in Elderly Psychiatric Patients and Healthy Elderly Individuals. *Journal of Geriatric Psychiatry and Neurology.* 2001;14(1):11-16. https://doi.org/10.1177/089198870101400104
114. Lampe IK, Sitskoorn MM, Heeren TJ. Effects of recurrent major depressive disorder on behavior and cognitive function in female depressed patients. *Psychiatry Research.* 2004;125(2):73-79. https://doi.org/10.1016/j.psychres.2003.12.004
115. Lansdall CJ, Coyle-Gilchrist ITS, Jones PS, et al. Apathy and impulsivity in frontotemporal lobar degeneration syndromes. *Brain.* 2017;140(6):1792-1807. https://doi.org/10.1093/brain/awx101
116. Lawson RP, Nord CL, Seymour B, et al. Disrupted habenula function in major depression. *Molecular Psychiatry.* 2017;22:202-208. https://doi.org/10.1038/mp.2016.81
117. Lemke MR, Schleidt M. Temporal segmentation of human short-term behavior in everyday activities and interview sessions. *Naturwissenschaften.* 1999;86(6):289-292. https://doi.org/10.1007/s001140050617
118. Lemke MR. Effect of reboxetine on depression in Parkinson's disease patients. *The Journal of clinical psychiatry.* 2002;63(4):300-304.
119. Lemke MR, Brecht HM, Koester J, Kraus PH, Reichmann H. Anhedonia, Depression, and Motor Functioning in Parkinson's Disease During Treatment With Pramipexole. *The Journal of Neuropsychiatry and Clinical Neurosciences.* 2005;17(2):214-220. https://doi.org/10.1176/jnp.17.2.214
120. Lemke MR, Koethe NH, Schleidt M. Timing of movements in depressed patients and healthy controls. *Journal of Affective Disorders.* 1999;56(2-3):209-214. https://doi.org/10.1016/S0165-0327(99)00034-8
121. Lemke MR, Puhl P, Broderick A. Motor activity and perception of sleep in depressed patients. *Journal of Psychiatric Research.* 1999;33(3):215-224. https://doi.org/10.1016/S0022-3956(98)00067-3
122. Lemke MR, Wendorff T, Mieth B, Buhl K, Linnemann M. Spatiotemporal gait patterns during over ground locomotion in major depression compared with healthy controls. *Journal of Psychiatric Research.* 2000;34(4-5):277-283. https://doi.org/10.1016/S0022-3956(00)00017-0

© 2020 Trøstheim M et al. *JAMA Network Open.*

123. Lempert KM, Pizzagalli DA. Delay discounting and future-directed thinking in anhedonic individuals. *Journal of Behavior Therapy and Experimental Psychiatry.* 2010;41(3):258-264. https://doi.org/10.1016/j.jbtep.2010.02.003
124. Lewandowski KE, Whitton AE, Pizzagalli DA, Norris LA, Ongur D, Hall M-H. Reward learning, neurocognition, social cognition, and symptomatology in psychosis. *Frontiers in psychiatry.* 2016;7:100. https://doi.org/10.3389/fpsyt.2016.00100
125. Lin Y-F, Chen C-Y, Ongur D, et al. Polygenic pleiotropy and potential causal relationships between educational attainment, neurobiological profile, and positive psychotic symptoms. *Translational Psychiatry.* 2018;8. https://doi.org/10.1038/s41398-018-0144-4
126. Liu W-h, Chan RCK, Wang L-z, et al. Deficits in sustaining reward responses in subsyndromal and syndromal major depression. *Progress in Neuro-Psychopharmacology and Biological Psychiatry.* 2011;35(4):1045-1052. https://doi.org/10.1016/j.pnpbp.2011.02.018
127. Liu W-h, Wang L-z, Shang H-r, et al. The influence of anhedonia on feedback negativity in major depressive disorder. *Neuropsychologia.* 2014;53:213-220. https://doi.org/10.1016/j.neuropsychologia.2013.11.023
128. Liu W-h, Roiser JP, Wang L-z, et al. Anhedonia is associated with blunted reward sensitivity in first-degree relatives of patients with major depression. *Journal of Affective Disorders.* 2016;190:640-648. https://doi.org/10.1016/j.jad.2015.10.050
129. Liu W-H, Valton V, Wang L-Z, Zhu Y-H, Roiser JP. Association between habenula dysfunction and motivational symptoms in unmedicated major depressive disorder. *Social Cognitive and Affective Neuroscience.* 2017;12(9):1520-1533. https://doi.org/10.1093/scan/nsx074
130. Liu W-h, Wang L-z, Zhao S-h, Ning Y-p, Chan RCK. Anhedonia and emotional word memory in patients with depression. *Psychiatry Research.* 2012;200(2-3):361-367. https://doi.org/10.1016/j.psychres.2012.07.025
131. Liu W-h, Wang L-z, Zhu Y-h, Li M-h, Chan RCK. Clinical utility of the Snaith-Hamilton-Pleasure scale in the Chinese settings. *BMC Psychiatry.* 2012;12(1):184. https://doi.org/10.1186/1471-244X-12-184
132. Loas G, Monestes JL, Ameller A, et al. Traduction et étude de validation de la version française de l'échelle d'expérience temporelle du plaisir (EETP, Temporal Experience of Pleasure Scale [TEPS], Gard et al., 2006) : étude chez 125 étudiants et chez 162 sujets présentant un trouble psychiatrique [Psychometric properties of the French version of the Temporal Experience of Pleasure Scale (TEPS): Study on 125 university students and on 162 psychiatric subjects]. *Annales Médico-psychologiques, revue psychiatrique.* 2009;167(9):641-648. https://doi.org/10.1016/j.amp.2009.09.002
133. Loas G, Duru C, Godefroy O, Krystkowiak P. Hedonic deficits in Parkinson's disease: is consummatory anhedonia specific? *Frontiers in Neurology.* 2014;5:24. https://doi.org/10.3389/fneur.2014.00024
134. Lorenzini N. *The relevance and usefulness of the Implicit Associations Test (IAT) in adolescent development, attachment and depression.* [Doctoral thesis], University College London; 2015.
135. Mann CL, Footer O, Chung YS, Driscoll LL, Barch DM. Spared and impaired aspects of motivated cognitive control in schizophrenia. *Journal of abnormal psychology.* 2013;122(3):745-755. https://doi.org/10.1037/a003306
136. Martino I, Santangelo G, Moschella D, et al. Assessment of Snaith-Hamilton Pleasure Scale (SHAPS): the dimension of anhedonia in Italian healthy sample. *Neurological Sciences.* 2018;39(4):657-661. https://doi.org/10.1007/s10072-018-3260-2
137. Martinotti G, Cloninger CR, Janiri L. Temperament and Character Inventory Dimensions and Anhedonia in Detoxified Substance-Dependent Subjects. *The American Journal of Drug and Alcohol Abuse.* 2008;34(2):177-183. https://doi.org/10.1080/00952990701877078
138. Martinotti G, Di Nicola M, Reina D, et al. Alcohol Protracted Withdrawal Syndrome: The Role of Anhedonia. *Substance Use & Misuse.* 2008;43(3-4):271-284. https://doi.org/10.1080/10826080701202429
139. Matsui K, Tachibana H, Yamanishi T, et al. Clinical correlates of anhedonia in patients with Parkinson's disease. *Clinical Neurology and Neurosurgery.* 2013;115(12):2524-2527. https://doi.org/10.1016/j.clineuro.2013.10.013
140. Mazza M, Catalucci A, Pino MC, et al. Dysfunctional neural networks associated with impaired social interactions in early psychosis: an ICA analysis. *Brain Imaging and Behavior.* 2013;7(3):248-259. https://doi.org/10.1007/s11682-013-9223-6
141. McCabe C, Woffindale C, Harmer CJ, Cowen PJ. Neural Processing of Reward and Punishment in Young People at Increased Familial Risk of Depression. *Biological Psychiatry.* 2012;72(7):588-594. https://doi.org/10.1016/j.biopsych.2012.04.034

© 2020 Trøstheim M et al. *JAMA Network Open.*

142. Mies GW, de Water E, Wiersema JR, Scheres A. Delay discounting of monetary gains and losses in adolescents with ADHD: Contribution of delay aversion to choice. *Child Neuropsychology.* 2019;25(4):528-547. https://doi.org/10.1080/09297049.2018.1508563
143. Milders M, Bell S, Boyd E, et al. Reduced detection of positive expressions in major depression. *Psychiatry Research.* 2016;240:284-287. https://doi.org/10.1016/j.psychres.2016.04.075
144. Misaki M, Suzuki H, Savitz J, Drevets WC, Bodurka J. Individual Variations in Nucleus Accumbens Responses Associated with Major Depressive Disorder Symptoms. *Scientific Reports.* 2016;6:21227. https://doi.org/10.1038/srep21227
145. Morris LS, Kundu P, Costi S, et al. Ultra-high field MRI reveals mood-related circuit disturbances in depression: a comparison between 3-Tesla and 7-Tesla. *Translational Psychiatry.* 2019;9. https://doi.org/10.1038/s41398-019-0425-6
146. Mrochen A, Marxreiter F, Kohl Z, et al. From sweet to sweat: Hedonic olfactory range is impaired in Parkinson's disease. *Parkinsonism & Related Disorders.* 2016;22:9-14. https://doi.org/10.1016/j.parkreldis.2015.09.035
147. Nagayama H, Kubo S-i, Hatano T, et al. Validity and Reliability Assessment of a Japanese Version of the Snaith-Hamilton Pleasure Scale. *Internal Medicine.* 2012;51(8):865-869. https://doi.org/10.2169/internalmedicine.51.6718
148. Nagayama H, Maeda T, Uchiyama T, et al. Anhedonia and its correlation with clinical aspects in Parkinson's disease. *Journal of the Neurological Sciences.* 2017;372:403-407. https://doi.org/10.1016/j.jns.2016.10.051
149. Nakonezny PA, Morris DW, Greer TL, et al. Evaluation of anhedonia with the Snaith–Hamilton Pleasure Scale (SHAPS) in adult outpatients with major depressive disorder. *Journal of Psychiatric Research.* 2015;65:124-130. https://doi.org/10.1016/j.jpsychires.2015.03.010
150. Ng CG, Chin SC, Yee AHA, et al. Validation of Malay Version of Snaith-Hamilton Pleasure Scale: Comparison between Depressed Patients and Healthy Subjects at an Out-Patient Clinic in Malaysia. *The Malaysian journal of medical sciences : MJMS.* 2014;21(3):62-70.
151. Norbury A, Kurth-Nelson Z, Winston JS, Roiser JP, Husain M. Dopamine Regulates Approach-Avoidance in Human Sensation-Seeking. *International Journal of Neuropsychopharmacology.* 2015;18(10):pyv041. https://doi.org/10.1093/ijnp/pyv041
152. Nord CL, Lawson RP, Huys QJM, Pilling S, Roiser JP. Depression is associated with enhanced aversive Pavlovian control over instrumental behaviour. *Scientific Reports.* 2018;8. https://doi.org/10.1038/s41598-018-30828-5
153. Nugent AC, Ballard ED, Gould TD, et al. Ketamine has distinct electrophysiological and behavioral effects in depressed and healthy subjects. *Molecular Psychiatry.* 2018. https://doi.org/10.1038/s41380-018-0028-2
154. Osuch EA, Manning K, Hegele RA, et al. Depression, marijuana use and early-onset marijuana use conferred unique effects on neural connectivity and cognition. *Acta Psychiatrica Scandinavica.* 2016;134(5):399-409. https://doi.org/10.1111/acps.12629
155. Pechtel P, Pizzagalli DA. Disrupted reinforcement learning and maladaptive behavior in women with a history of childhood sexual abuse: A high-density event-related potential study. *JAMA Psychiatry.* 2013;70(5):499-507. https://doi.org/10.1001/jamapsychiatry.2013.728
156. Pechtel P, Dutra SJ, Goetz EL, Pizzagalli DA. Blunted reward responsiveness in remitted depression. *Journal of Psychiatric Research.* 2013;47(12):1864-1869. https://doi.org/10.1016/j.jpsychires.2013.08.011
157. Peciña M, Sikora M, Avery ET, et al. Striatal dopamine D2/3 receptor-mediated neurotransmission in major depression: Implications for anhedonia, anxiety and treatment response. *European Neuropsychopharmacology.* 2017;27(10):977-986. https://doi.org/10.1016/j.euroneuro.2017.08.427
158. Pettorruso M, Martinotti G, Fasano A, et al. Anhedonia in Parkinson's disease patients with and without pathological gambling: A case-control study. *Psychiatry Research.* 2014;215(2):448-452. https://doi.org/10.1016/j.psychres.2013.12.013
159. Pizzagalli DA, Berretta S, Wooten D, et al. Assessment of striatal dopamine transporter binding in individuals with major depressive disorder: In vivo positron emission tomography and postmortem evidence. *JAMA Psychiatry.* 2019. https://doi.org/10.1001/jamapsychiatry.2019.0801
160. Polimeni JO, Campbell DW, Gill D, Sawatzky BL, Reiss JP. Diminished humour perception in schizophrenia: Relationship to social and cognitive functioning. *Journal of Psychiatric Research.* 2010;44(7):434-440. https://doi.org/10.1016/j.jpsychires.2009.10.003
161. Pomponi M, Loria G, Salvati S, et al. DHA effects in Parkinson disease depression. *Basal Ganglia.* 2014;4(2):61-66. https://doi.org/10.1016/j.baga.2014.03.004

© 2020 Trøstheim M et al. *JAMA Network Open.*

162. Pontieri FE, Assogna F, Pellicano C, et al. Sociodemographic, neuropsychiatric and cognitive characteristics of pathological gambling and impulse control disorders NOS in Parkinson's disease. *European Neuropsychopharmacology.* 2015;25(1):69-76. https://doi.org/10.1016/j.euroneuro.2014.11.006
163. Redlich R, Dohm K, Grotegerd D, et al. Reward Processing in Unipolar and Bipolar Depression: A Functional MRI Study. *Neuropsychopharmacology.* 2015;40:2623-2631. https://doi.org/10.1038/npp.2015.110
164. Renfroe JB, Bradley MM, Okun MS, Bowers D. Motivational engagement in Parkinson's disease: Preparation for motivated action. *International Journal of Psychophysiology.* 2016;99:24-32. https://doi.org/10.1016/j.ijpsycho.2015.11.014
165. Richards DA, Rhodes S, Ekers D, et al. Cost and Outcome of BehaviouRal Activation (COBRA): a randomised controlled trial of behavioural activation versus cognitive-behavioural therapy for depression. *Health Technology Assessment.* 2017;21(46):1-366. https://doi.org/10.3310/hta21460
166. Rizvi SJ. *Anhedonia in Major Depressive Disorder: Exploration of a Predictive Clinical Phenotype* [Doctoral thesis], University of Toronto; 2015.
167. Rothkirch M, Tonn J, Köhler S, Sterzer P. Neural mechanisms of reinforcement learning in unmedicated patients with major depressive disorder. *Brain.* 2017;140(4):1147-1157. https://doi.org/10.1093/brain/awx025
168. Ryu V. *Dysfunctional reward learning in bipolar disorder: An event-related potential study* [Doctoral thesis], Yonsei University; 2013.
169. Rzepa E, McCabe C. Decreased anticipated pleasure correlates with increased salience network resting state functional connectivity in adolescents with depressive symptomatology. *Journal of Psychiatric Research.* 2016;82:40-47. https://doi.org/10.1016/j.jpsychires.2016.07.013
170. Santangelo G, Morgante L, Savica R, et al. Anhedonia and cognitive impairment in Parkinson's disease: Italian validation of the Snaith–Hamilton Pleasure Scale and its application in the clinical routine practice during the PRIAMO study. *Parkinsonism & Related Disorders.* 2009;15(8):576-581. https://doi.org/10.1016/j.parkreldis.2009.02.004
171. Santangelo G, Vitale C, Trojano L, et al. Relationship between depression and cognitive dysfunctions in Parkinson's disease without dementia. *Journal of Neurology.* 2009;256(4):632-638. https://doi.org/10.1007/s00415-009-0146-5
172. Scheidegger M, Walter M, Lehmann M, et al. Ketamine Decreases Resting State Functional Network Connectivity in Healthy Subjects: Implications for Antidepressant Drug Action. *PLOS ONE.* 2012;7(9):e44799. https://doi.org/10.1371/journal.pone.0044799
173. Schneier FR, Slifstein M, Whitton AE, et al. Dopamine Release in Antidepressant-Naive Major Depressive Disorder: A Multimodal C-11 -(+)-PHNO Positron Emission Tomography and Functional Magnetic Resonance Imaging Study. *Biological Psychiatry.* 2018;84(8):563-573. https://doi.org/10.1016/j.biopsych.2018.05.014
174. Sobesky EN. *Activación neural asociada a interacciones sociales en la depresión mayor: Potenciales relacionados a eventos durante el ultimatum game*, University of the Republic; 2017.
175. Spalletta G, Fagioli S, Meco G, et al. Hedonic tone and its mood and cognitive correlates in Parkinson's disease. *Depression and Anxiety.* 2013;30(1):85-91. https://doi.org/10.1002/da.22036
176. Spalletta G, Robinson RG, Cravello L, et al. The early course of affective and cognitive symptoms in de novo patients with Parkinson's disease. *Journal of neurology.* 2014;261(6):1126-1132. https://doi.org/10.1007/s00415-014-7327-6
177. Sprengelmeyer R, Steele JD, Mwangi B, et al. The insular cortex and the neuroanatomy of major depression. *Journal of Affective Disorders.* 2011;133(1-2):120-127. https://doi.org/10.1016/j.jad.2011.04.004
178. Steele JD, Kumar P, Ebmeier KP. Blunted response to feedback information in depressive illness. *Brain.* 2007;130(9):2367-2374. https://doi.org/10.1093/brain/awm150
179. Stevens A, Peschk I, Schwarz J. Implicit learning, executive function and hedonic activity in chronic polydrug abusers, currently abstinent polydrug abusers and controls. *Addiction.* 2007;102(6):937-946. https://doi.org/10.1111/j.1360-0443.2007.01823.x
180. Stroud JB, Freeman TP, Leech R, et al. Psilocybin with psychological support improves emotional face recognition in treatment-resistant depression. *Psychopharmacology.* 2018;235(2):459-466. https://doi.org/10.1007/s00213-017-4754-y

181. Szczepanik JE, Furey ML, Nugent AC, Henter ID, Zarate CA, Lejuez CW. Altered interaction with environmental reinforcers in major depressive disorder: Relationship to anhedonia. *Behaviour Research and Therapy.* 2017;97:170-177. https://doi.org/10.1016/j.brat.2017.08.003
182. Taalman HC. *The Effects of Transcranial Magnetic Stimulation on Depression and the Associated Olfactory Dysfunction* [Master thesis], Queen's University; 2017.
183. Taubitz LE. *Facilitating Visual Selective Attention via Monetary Reward: the Influence of Feedback, Hedonic Capacity, and Lifetime Major Depressive Disorder* [Doctoral thesis], University of Wisconsin-Milwaukee; 2015.
184. Tonioni F, Mazza M, Autullo G, et al. Is Internet addiction a psychopathological condition distinct from pathological gambling? *Addictive Behaviors.* 2014;39(6):1052-1056. https://doi.org/10.1016/j.addbeh.2014.02.016
185. Tremblay LK, Naranjo CA, Cardenas L, Herrmann N, Busto UE. Probing brain reward system function in major depressive disorder: Altered response to dextroamphetamine. *Archives of General Psychiatry.* 2002;59(5):409-416. https://doi.org/10.1001/archpsyc.59.5.409
186. Tremblay LK, Naranjo CA, Graham SJ, et al. Functional neuroanatomical substrates of altered reward processing in major depressive disorder revealed by a dopaminergic probe. *Archives of General Psychiatry.* 2005;62(11):1228-1236. https://doi.org/10.1001/archpsyc.62.11.1228
187. Tudge L, Williams C, Cowen PJ, McCabe C. Neural Effects of Cannabinoid CB1 Neutral Antagonist Tetrahydrocannabivarin on Food Reward and Aversion in Healthy Volunteers. *International Journal of Neuropsychopharmacology.* 2015;18(6):pyu094. https://doi.org/10.1093/ijnp/pyu094
188. Ubl B, Kuehner C, Kirsch P, Ruttorf M, Diener C, Flor H. Altered neural reward and loss processing and prediction error signalling in depression. *Social Cognitive and Affective Neuroscience.* 2015;10(8):1102-1112. https://doi.org/10.1093/scan/nsu158
189. Versace F, Frank DW, Stevens EM, Deweese MM, Guindani M, Schembre SM. The reality of "food porn": Larger brain responses to food-related cues than to erotic images predict cue-induced eating. *Psychophysiology.* 2019;56(4). https://doi.org/10.1111/psyp.13309
190. Vidotto G, Catalucci A, Roncone R, Pino MC, Mazza M. Neural correlates of observation of disgusting images in subjects with first episode psychosis and post-traumatic stress disorder. *Journal of Biological Regulators and Homeostatic Agents.* 2014;28(4):705-716.
191. Vrieze E, Ceccarini J, Pizzagalli DA, et al. Measuring extrastriatal dopamine release during a reward learning task. *Human Brain Mapping.* 2013;34(3):575-586. https://doi.org/10.1002/hbm.21456
192. Vrieze E, Pizzagalli DA, Demyttenaere K, et al. Reduced Reward Learning Predicts Outcome in Major Depressive Disorder. *Biological Psychiatry.* 2013;73(7):639-645. https://doi.org/10.1016/j.biopsych.2012.10.014
193. Walsh AE, Browning M, Drevets WC, Furey M, Harmer CJ. Dissociable temporal effects of bupropion on behavioural measures of emotional and reward processing in depression. *Philosophical Transactions of the Royal Society B.* 2018;373(1742):20170030. https://doi.org/10.1098/rstb.2017.0030
194. Walsh AEL, Huneke NTM, Brown R, Browning M, Cowen P, Harmer CJ. A Dissociation of the Acute Effects of Bupropion on Positive Emotional Processing and Reward Processing in Healthy Volunteers. *Frontiers in Psychiatry.* 2018;9. https://doi.org/10.3389/fpsyt.2018.00482
195. Wardle MC, Vincent JN, Suchting R, Green CE, Lane SD, Schmitz JM. Anhedonia Is Associated with Poorer Outcomes in Contingency Management for Cocaine Use Disorder. *Journal of Substance Abuse Treatment.* 2017;72:32-39. https://doi.org/10.1016/j.jsat.2016.08.020
196. Witt K, Daniels C, Reiff J, et al. Neuropsychological and psychiatric changes after deep brain stimulation for Parkinson's disease: a randomised, multicentre study. *The Lancet Neurology.* 2008;7(7):605-614. https://doi.org/10.1016/S1474-4422(08)70114-5
197. Yang X-h, Wang Y, Wang D-f, et al. White matter microstructural abnormalities and their association with anticipatory anhedonia in depression. *Psychiatry Research: Neuroimaging.* 2017;264:29-34. https://doi.org/10.1016/j.pscychresns.2017.04.005
198. Yoshida K, Shimizu Y, Yoshimoto J, et al. Prediction of clinical depression scores and detection of changes in whole-brain using resting-state functional MRI data with partial least squares regression. *PLOS ONE.* 2017;12(7):e0179638. https://doi.org/10.1371/journal.pone.0179638
199. Young KD, Bellgowan PSF, Bodurka J, Drevets WC. Behavioral and neurophysiological correlates of autobiographical memory deficits in patients with depression and individuals at high risk for depression. *JAMA Psychiatry.* 2013;70(7):698-708. https://doi.org/10.1001/jamapsychiatry.2013.1189

© 2020 Trøstheim M et al. *JAMA Network Open.*

200. Young KD, Siegle GJ, Zotev V, et al. Randomized Clinical Trial of Real-Time fMRI Amygdala Neurofeedback for Major Depressive Disorder: Effects on Symptoms and Autobiographical Memory Recall. *American Journal of Psychiatry.* 2017;174(8):748-755. https://doi.org/10.1176/appi.ajp.2017.16060637
201. Young KD, Bellgowan PSF, Bodurka J, Drevets WC. Neurophysiological correlates of autobiographical memory deficits in currently and formerly depressed subjects. *Psychological Medicine.* 2014;44(14):2951-2963. https://doi.org/10.1017/S0033291714000464
202. Yuan H, Phillips R, Wong CK, et al. Tracking resting state connectivity dynamics in veterans with PTSD. *Neuroimage-Clinical.* 2018;19:260-270. https://doi.org/10.1016/j.nicl.2018.04.014
203. Zahodne LB, Marsiske M, Okun MS, Bowers D. Components of Depression in Parkinson Disease. *Journal of Geriatric Psychiatry and Neurology.* 2012;25(3):131-137. https://doi.org/10.1177/0891988712455236
204. Abramovitch A, Pizzagalli DA, Reuman L, Wilhelm S. Anhedonia in obsessive-compulsive disorder: Beyond comorbid depression. *Psychiatry Research.* 2014;216(2):223-229. https://doi.org/10.1016/j.psychres.2014.02.002
205. Olson EA, Kaiser RH, Pizzagalli DA, Rauch SL, Rosso IM. Anhedonia in Trauma-Exposed Individuals: Functional Connectivity and Decision-Making Correlates. *Biological Psychiatry: Cognitive Neuroscience and Neuroimaging.* 2018;3(11):959-967. https://doi.org/10.1016/j.bpsc.2017.10.008

© 2020 Trøstheim M et al. *JAMA Network Open.*
